# Health assessment of non-native red-eared sliders (*Trachemys scripta elegans*) and their impact potential on native species

**DOI:** 10.1371/journal.pone.0333786

**Published:** 2025-10-06

**Authors:** John M. Winter, Kaitlin Moorhead, Kamila Grochowski-Grum, Chris Anchor, Jennifer A. Landolfi, Laura A. Adamovicz, Matthew C. Allender

**Affiliations:** 1 University of Illinois Wildlife Epidemiology Laboratory, Urbana, Illinois, United States of America; 2 Forest Preserve District of Cook County, River Forest, Illinois, United States of America; 3 Brookfield Zoo Chicago, Brookfield, Illinois, United States of America; 4 Zoological Pathology Program, University of Illinois, Brookfield, Illinois, United States of America; 5 Veterinary Diagnostic Laboratory, University of Illinois College of Veterinary Medicine, Urbana, Illinois, United States of America; University of Missouri in Columbia, BRAZIL

## Abstract

Red-eared sliders (RES; *Trachemys scripta elegans*) are a globally invasive species that can impact native chelonian populations through resource competition and disease introduction. In Cook County, Illinois, invasive RES co-occur with, and greatly outnumber, a species of conservation priority in the Great Lakes region: the Blanding’s turtle (*Emydoidea blandingii*). This study sampled free-ranging RES in Cook County during spring, summer, and fall in four unique locations to characterize the possible effect of this non-native species on the health of regional, sympatric, aquatic chelonians. RES (n = 242) were captured and sampled for qPCR pathogen detection, clinical pathology, and necropsy from 2018 to 2022. Multiple pathogens were detected, including *Mycoplasma* spp., multiple adenoviruses, *Trachemys* herpesvirus 1 (TrHV-1), frog virus 3, human-pathogenic *Leptospira* spp., *Emydomyces testavorans*, and *Salmonella typhimurium*. *Mycoplasma* spp. prevalence was significantly higher (p < 0.05) at one isolated site with a suspected greater density of turtles. Detection of TrHV-1 was significantly associated with season (p < 0.001), with detections occurring only in spring and fall. For the 28 hematology, plasma biochemistry, and protein electrophoresis analytes assayed, significant and variable associations occurred based on sample year, season, pathogen detection, age, and sex. Population-based reference intervals were created for hematologic, plasma biochemistry, and plasma protein electrophoresis analytes. Common necropsy findings included mild endoparasitism and related vascular lesions such as endarteritis. Notably, qPCR detection of above pathogens was not associated with any gross or histologic lesions indicative of clinically relevant disease. This study indicates that invasive RES in Cook County may be infected with pathogens of concern for co-occurring turtle species, and absence of associated lesions in the RES suggests they likely serve as carrier species for these pathogens. Continued health monitoring of this species is important as interactions with chelonians of conservation priority increase.

## Introduction

Red-eared sliders (RES; *Trachemys scripta elegans*) are medium-sized aquatic chelonians that are listed as one of the world’s most detrimental invasive species [1]. Their native range includes the Southern, Eastern, and Midwestern portions of the United States and they are considered non-native in Northeastern Illinois [2]. Through competition for food and basking spots, invasive RES have been proposed to directly reduce the fitness of native turtles [3–5]. Blanding’s turtles (*Emydoidea blandingii*) are medium-sized semi-aquatic chelonians that are listed as state-endangered in Illinois and a species of conservation priority in the Great Lakes region [6,7]. The effects of red-eared slider presence on Blanding’s turtle populations have not been assessed but models predict an increase in red-eared-slider-suitable-habitat from 26% to 39–50% of the entire Great Lakes basin by 2050 and suggest increased future interaction and potential competition between the two species [7].

Beyond population-level consequences due to resource competition, RES are also well-documented pathogen reservoirs. Free-ranging RES are hosts to various pathogens of concern to other chelonians they live with and/or humans in non-native ranges, including: *Salmonella* spp. in China [8,9], *Fusarium* spp. in Spain [10], platyhelminths in France [11], *Chlamydia* spp., and *Cryptosporidium parvum* in Poland [12,13]. Other pathogens that have been detected in free-ranging RES include *Trachemys* herpesvirus 1 (TrHV-1) in New York, USA [14], *Leptospira* spp. in Italy [15], and *Mycoplasma* spp. in California, USA and Spain [16,17]. Through experimental study and surveillance, RES have also been identified as a potential reservoir for ranaviruses (family Iridoviridae), which have previously been associated with mass mortality events in free-ranging chelonians [18–21]. Several pathogen classes shared by RES have previously been detected in Blanding’s turtles, including: herpesviruses, *Salmonella* Typhimurium, *Mycoplasma* spp., and *Leptospira* spp. [22–24]. Invasion of RES into Blanding’s turtle habitat may lead to exposure of naïve Blanding’s turtles to co-invading pathogens. Additionally, the introduction of invasive alien species to non-native ranges can also act as an amplifying host or cause spillback of endemic pathogens to native hosts [25]. Therefore, pathogen surveillance in both of these species along borders of the RES geographic distribution are critical for identification of pathogen spillover events and resultant disease outbreaks.

Characterizing the health and pathogen profile of wild turtles has previously been accomplished through molecular testing of swab and tissue samples for pathogens, hematology such as complete blood counts and plasma biochemistry, and post-mortem examination with histopathology [24,26–28]. To allow for hematologic analysis, reference intervals that can be species-, location-, and individual specific are useful to determine which turtles fall outside of expected values for markers of inflammation, infection, or other disease [28]. Currently, there are limited hematologic reference intervals available for free-ranging RES including a series of captive animals [29,30] and a population of free-ranging invasive RES sampled during Fall in Germany [31].

Cook County, IL represents another border of RES invasion; historical surveys indicate that RES are likely an introduced species in this location [2]. Threats to Blanding’s turtles in the Chicago area are similar to other regions including a low reproductive output and delayed sexual maturity life history strategy, habitat fragmentation and degradation, high rates of hatchling and nest predation, and road mortality [32–35]. The addition of any further threats, such as RES-driven competition or disease, could be detrimental to population persistence. Therefore, the purposes of the current study were to 1) perform pathogen surveillance via detection of DNA from known and novel pathogens in red-eared sliders, 2) report reference intervals for clinical pathology parameters in apparently healthy free-ranging red-eared sliders, and 3) report common necropsy findings for red-eared sliders following population management through removal.

## Materials and methods

### Field techniques

Free-ranging RES were sampled from 2018 to 2022 at three seasonal time points (spring, summer, and fall) during the months of May through October. Other turtles captured were either released immediately or sampled for other projects. More specifically, RES were sampled in July, 2018; June and July of 2019; June and September of 2020; May, August, October of 2021; and May, June, August, September, and October of 2022. Spring was considered May – June whereas summer was July through September and fall was October.

RES were found in Cook County, IL at eight sites (Sites A – H) which contained a mixture of wetlands, forest, riverine and upland eco-types. Sites A and B are marsh habitats with ephemeral swales and nearby forested habitat. Sites C – E are upland, forested habitats with a large river system flowing through them and small pools branching off of this river system. Sites F – H are large, deep lakes used primarily for public fishing. RES and Blanding’s turtles are known to co-inhabit one of these sites (Site A), while RES co-occur with a number of common chelonian species (e.g., *Sternotherus odoratus*, *Chelydra serpentina*, *Chrysemys picta*, *Graptemys geographica*, *Apalone spinifera*) variably in all other sites.

All RES were captured using double-throated hoop traps and sampled following approved procedures in the Protocol 20258 approved by the University of Illinois’ Institutional Animal Care and Use Committee. From 2018–2020, turtles were removed from the field for sampling prior to euthanasia or release. In 2021 and 2022, turtles were sampled in the field and released on-site or removed for euthanasia by the Forest Preserves of Cook County under Illinois Department of Natural Resources Scientific Collectors Permit: HSCP19−16.

### Sampling protocol

Trapping effort and the components of RES health assessments (i.e., physical exam, hematology, plasma biochemistry, protein electrophoresis, computed tomography (CT), and necropsy) differed between years.

At the time of collection in all years of the study, every turtle was 1) assigned an age class (juvenile or adult) based on total plastron length (adult males >100 mm, adult females >160 mm), 2) assigned a sex (male, female, unknown) based on plastron concavity, foreclaw length, and tail length, and 3) permanently marked via marginal scute notching [36–38]. Beginning in 2021, each turtle also received a complete physical exam consisting of evaluation of all external anatomy for any observable abnormalities, such as recording nasal discharge, oral ulcers/plaques, and nare asymmetry as signs of possible upper respiratory disease.

Beginning in 2019, combined oral-cloacal (COS, 2019–2021) or oral-cloacal-shell swabs (COSS, 2022) were collected from each turtle using cotton-tipped plastic-handled applicators (Fisher Scientific, Pittsburgh, Pennsylvania 15275, USA) and placed on ice packs until transport to the laboratory two to six hours later. Swabs were then stored at −20°C or −80°C until DNA extraction.

Beginning in 2021, blood was collected from the subcarapacial sinus using a 22- or 25-gauge needle and syringe. Total blood volume collected did not exceed 0.8% of total body weight. Blood was immediately stored in lithium heparin blood tubes (BD microtainer, Jorgensen Laboratories Inc, Loveland, CO 80538) and placed on ice packs until transport to laboratory. Following same-day laboratory analysis, whole blood and plasma samples were stored at −20°C or −80°C, respectively.

### Clinical pathology

In 2021 and 2022, same-day laboratory analysis was performed by measuring packed cell volume (PCV) and total solids (TS) as previously described, and performing a total white blood cell (WBC) count using Avian Leukopets (Vet Lab Supply, Palmetto Bay, FL, USA) on Bright-line hemacytometers (Hausser Scientific, Horsham, PA, USA) following the manufacturer’s protocol [28]. Fresh blood smears were stained with a modified Wright-Giemsa stain and one hundred white blood cell differential counts were performed by a single observer (KM). Plasma biochemistry profiles were performed by the University of Illinois’ Veterinary Diagnostic Laboratory for the following analytes: total calcium, phosphorus, bile acids, uric acid, creatine kinase, aspartate aminotransferase (AST), sodium, chloride, glutamate dehydrogenase (GLDH), glucose, and potassium (AU680 Chemistry System, Beckman Coulter, Brea, CA 92821 USA).

Plasma protein electrophoresis was performed at the University of Miami Miller School Acute Phase Protein Lab according to the procedure provided by the Helena SPIFE 3000 system with the use of split beta gels (Helena Laboratories, Inc., Beaumont, Texas 77707, USA). Results were produced after gel scanning and analysis by Helena software. Fraction delimits were placed as previously demonstrated for Blanding’s turtles [39]. Percentages for each fraction were determined by this software, and absolute values (g/dl) for each fraction were obtained by multiplying the percentage by the total protein (TP) concentration. The albumin:globulin (A:G) ratio was calculated by dividing albumin by the sum of the globulin fractions.

### Pathogen testing

DNA was extracted from swabs using a commercially available kit (Qiagen DNeasy; Qiagen, Valencia, California 91355, USA) following manufacturer’s protocol. Extractions from COSS swabs were modified by adding a lyticase digestion (300 units at 37°C for 1 hour) prior to the lysis step to increase the recovery of fungal DNA. Quantity (ng/µl) and quality (A260:A280 ratio) of DNA was evaluated using a Nanodrop spectrophotometer (Thermo Fisher Scientific Inc., Waltham, Massachusetts 02451, USA). Samples with A260:A280 greater than 2.4 were purified using a QIAquick PCR Purification Kit following manufacturers protocol [40].

Quantitative PCR (qPCR) was performed using a Fluidigm platform with associated software (Fluidigm, South San Francisco, California 94080, USA) to assay for 4–18 pathogens, depending on the year, funding available, and primer availability, using published or in-house primer-probe assays as previously described (Table 1) [41]. Briefly, all samples and standards were evaluated in triplicate and a non-template control was included on each plate. Each sample’s cycle threshold (CT) value was compared to a standard curve consisting of serial dilutions from 10^1^–10^7^ copies of each gene target. Copy numbers were normalized based on starting DNA concentration, and final results were reported as copies/ng DNA. A standard curve was not available for *S. typhimurium* or *S. enteritidis*, so a Fluidigm CT cutoff value of 24 was used to distinguish positive and negative samples. All Fluidigm positive samples were confirmed on a simplex assay using the same primer set on a QuantStudio 3 (ThermoFisher Scientific) with full standard curve and non-template control.

**Table 1:** Pathogens tested for in red-eared sliders (*Trachemys scripta elegans*) between 2019 and 2022 in Cook County, IL [42–54].

Years Tested				Pathogen	Primer source
2019	2020	2021	2022		
X	X	X	X	FV3 (ranavirus)	[42] Allender et al., 2013
X	X		X	*Ambystoma tigrinum* virus (ranavirus)	[48] Pallister et al., 2007
X	X		X	Bohle iridovirus (ranavirus)	[48] Pallister et al., 2007
X	X		X	Epizootic hematopoietic necrosis virus (ranavirus)	[48] Pallister et al., 2007
X	X		X	Pan-ranavirus	[51] Stilwell et al., 2018
X	X	X	X	*Emydomyces testavorans*	[54] Woodburn et al., 2019
X	X		X	*Emydoidea blandingii herpesvirus 1*	[46] Lindemann et al., 2018
			X	*Trachemys* herpesvirus 1	In house
X	X		X	Emydid herpesvirus 1	In house
X				Testudinid herpesvirus 2	[44] Braun et al., 2014
X	X		X	*Salmonella tymphimurium*	[49] Park et al., 2008
X	X		X	*Salmonella enteritidis*	[45] Levin, 2009
X	X		X	Intranuclear coccidia of Testudines (TINC)	[43] Alvarez et al., 2013
	X		X	Human-pathogenic *Leptospira* spp.	[50] Smythe et al., 2002
X	X		X	*Mycoplasma agassizii*	[44] Braun et al., 2014
X	X		X	*Mycoplasma testudineum*	[44] Braun et al., 2014
X	X		X	Box Turtle *Mycoplasmopsis* sp.	In house
X	X	X	X	Consensus *Mycoplasma*	[47] Ossiboff et al., 2015
			X	Consensus adenovirus	[53] Wellehan et al., 2004
		X		Consensus herpesvirus	[52] VanDevanter et al., 1996

Conventional consensus PCR (cPCR) was performed variably for adenoviruses, herpesviruses, and *Mycoplasma* spp. depending on the year, inclusive of positive and negative controls. PCR products producing appropriately-sized bands were purified using ExoSAP-IT (USB Corporation, Cleveland, Ohio 44128, USA), commercially sequenced (ACGT Inc., Wheeling, Illinois 60090, USA) in both directions, and compared to existing sequences in GenBank using BLASTn.

### Postmortem imaging and necropsy

For each sampling season (spring, summer, fall), up to seven RES were selected for humane euthanasia and necropsy. Turtles were selected based on the presence of abnormal clinical signs or based on location, with sites thought to be shared by Blanding’s turtles prioritized. Euthanasia was performed using propofol to induce anesthesia, followed by intravenous potassium chloride injection, then decapitation to ensure death. Turtles were imaged post-mortem via computed tomography (CT) performed with a Toshiba Aquilion LB (Large Bore) 16-slice CT scanner and the presence of shell lesions and ovarian follicles or eggs were recorded by a single observer (JW). Necropsy, with gross examination and complete histopathology, was performed by the University of Illinois’ Zoological Pathology Program.

### Statistical analysis

All statistical analysis was performed in R version 2023.12.1 Normality was assessed using histograms and the Shapiro-Wilk test set at alpha level = 0.05. Pathogen prevalence and 95% confidence intervals were calculated using a 1-sample proportions test without continuity correction. Statistical associations using any test were only assessed for pathogens with more than 5 detections over the course of the entire study. Case-wise deletion was performed for turtles missing data for predictor or response variables considered during statistical modeling to facilitate model ranking using an information-theoretic approach.

PCR pathogen detection status (positive or negative) was modeled using multivariable logistic regression models using the glm function in the “stats” package version 4.3.2. Models of fixed effects were considered separately (ex. a model of Season was created alone) and certain multivariate models were created where appropriate (ex. a model with Season and Location was created). A logit link function in the binomial family was used for glm models. Fixed effects considered were sites in which more than 4 turtles were tested (Sites A – D), season (spring, summer, fall), sex (female, male, unknown), year for *Mycoplasma* spp. and frog-virus 3 (FV3; 2021 and 2022), and age class (adult, juvenile). Candidate sets of models were ranked using Akaike’s information criterion (AIC) to identify the most parsimonious model predicting the detection of each pathogen (AICcmodavg package) [55].

Clinical pathology data were modeled using linear models, with the lm function, with fixed effects including pathogen detection status (TrHV-1, adenovirus, *Mycoplasma* spp.), age class, location, sex, season and year. As testing for TrHV-1 and adenoviruses was only performed in 2022, two separate groups of models were created with model group 1 containing data from 2021 and 2022 (TrHV-1 and adenovirus excluded from fixed effects) and model group 2 containing only data from 2022 (TrHV-1 and adenovirus included in fixed effects). Model ranking was performed separately for the model group 1 and model group 2 datasets using AIC and findings from the top-performing models predicting each clinical pathology parameter are reported.

Fisher’s Exact tests were used to identify associations between clinical signs and TrHV-1, FV3, and *Mycoplasma* spp. status. Clinical signs or other factors evaluated were those in which there were more than five occurrences and included: nares symmetry, appendage abnormalities, and the comorbidity of ectoparasite presence (leeches). A Fisher’s Exact test was also used to evaluate whether RES selected for euthanasia and pathologic assessment had differing pathogen detection rates compared to individuals released following sampling.

The relationship between radiographic findings identified via CT scan (i.e., gravidity, boney abnormalities of the shell) and clinical pathology parameters was assessed using t-tests for normally distributed data and Mann-Whitney U tests for non-normally distributed data. Descriptive statistics including counts and proportions were tabulated for imaging, gross and histopathologic abnormalities.

### Reference intervals

Population-based reference intervals were created for RES clinical pathology values as previously described [28,56]. Normality for each analyte was assessed via histogram, Q-Q plots, and Shapiro-Wilk tests with alpha = 0.05. Outliers were identified via the Horn method and 95% reference intervals were calculated with 90% lower and upper bound confidence intervals [57]. Turtles were excluded from analysis if they 1) were detected with any pathogens, 2) did not have clinical pathology testing performed, 3) did not have complete physical examinations, or 4) had clinically active abnormalities more severe than shell flaking/pitting, suspected congenital/developmental shell malformations, or non-active/healed lesions.

## Results

### Field sampling population

Between 2019 and 2022, 222 RES were sampled over 244 sampling events (Table 2). In 2021, the most common physical examination abnormalities were asymmetrical nares (n = 22), *Placobdella* spp. leech parasitism (n = 11), and shell abnormalities (n = 37) including discoloration, flaking, erosion, pitting, and fractures. In 2022, the most common abnormalities being asymmetrical nares (n = 14), *Placobdella* spp. leech parasitism (n = 27), and shell abnormalities (n = 99) including discoloration, flaking, erosion, pitting, and fractures.

**Table 2:** Health assessment tests performed for red-eared sliders (*Trachemys scripta elegans*) by year between 2018 and 2022 in Cook County, IL. Total number of samplings includes re-sampling of individuals that were recaptured during different seasons.

Tests	Years				
	2018	2019	2020	2021	2022
Total unique individuals sampled	10	24	20	84	104
Total number of samplings	10	24	20	101	109
Males	–	–	–	48	55
Females	–	–	–	44	48
Unknown Sex	–	–	–	9	6
Adult	–	–	–	89	103
Juvenile	–	–	–	12	6
Spring	–	–	–	35	40
Summer	10	24	20	34	35
Fall	–	–	–	32	34
Physical exam	0	0	0	60	108
Complete blood counts	0	0	0	83	105
Plasma biochemistry	0	0	0	92	92
Protein electrophoresis	0	0	0	0	104
Molecular pathogen testing	0	14	20	101	104
Computed tomography	0	0	0	21	21
Necropsy	10	24	20	21	21

### Pathogen detection

Two hundred and ten RES were tested for pathogens between 2019 and 2022. A total of 13 unique pathogens were detected via conventional and quantitative PCR at prevalences varying from 0.5–16.3% (Table 3). Co-detections occurred with Sulawesi tortoise adenovirus (TrHV-1: n = 1; *Leptospira* spp.: n = 1), a yellow-bellied slider adenovirus (*Salmonella typhimurium*: n = 1), an emydid *Mycoplasma* spp. (TrHV-1: n = 1), and FV3 (*Leptospira* spp.: n = 1).

**Table 3:** Pathogens detected via conventional PCR and real-time PCR in individual red-eared sliders (*Trachemys scripta elegans*) between 2019–2022 in Cook County, IL. Listed next to each pathogen are free-ranging North American emydid hosts in which previous detections have occurred.

Pathogen	Sequence for consensus PCR	N (positive/ total tested)	Prevalence	95% confidence interval	Previous free-ranging North American hosts
*Mycoplasma* sp.	^a^Emydid *Mycoplasma* sp.	17/ 210	8.1%	5.1–12.6%	red-eared slider [14], eastern box turtle [47]
	^b^Blanding’s turtle *Mycoplasma* sp.	1/ 210	0.5%	0.1–2.6%	Blanding’s turtle [24]
Herpesvirus	^c^Trachemys herpesvirus 1	1/ 84	1.2%	0.2–6.4%	red-eared slider [14]
Adenovirus	^d^Sulawesi tortoise adenovirus	8/ 92	8.7%	4.5–16.2%	Blanding’s turtle, red-eared slider, painted turtle [58,59]
	^**e**^Box turtle adenovirus	4/ 92	4.3%	1.7–10.7%	eastern box turtle [60]
	^**f**^Red-eared slider adenovirus	3/ 92	3.3%	1.1–9.2%	red-eared slider [61]
	^g^Yellow-bellied slider adenovirus 1	2/ 92	2.2%	0.6–7.6%	
	^h^Yellow-bellied slider adenovirus 2	1/ 92	1.1%	0.2–5.9%	
Trachemys herpesvirus 1	TaqMan qPCR	15/92	16.3%	10.1–25.2%	red-eared slider [14]
Frog virus 3	TaqMan qPCR	4/210	1.9%	0.7–4.8%	painted turtle [62], eastern box turtle [18], Florida box turtle (*Terrapene Carolina bauri*) [63], three-toed box turtle (*Terrapene mexicana triunguis*) [64], common snapping turtle [65], eastern mud turtle (*Kinosternon subrubrum*) [66]
*Leptospira* spp.	TaqMan qPCR	2/126	1.6%	0.4–5.6%	Blanding’s turtle [23]
*Emydomyces testavorans*	TaqMan qPCR	1/210	0.5%	0.1–2.6%	red-eared slider [67], western pond turtle (*Actinemys marmorata*), coastal plain cooter (*Pseudemys floridana*) [68]
*Salmonella* Typhimurium	TaqMan qPCR	1/126	0.8%	0.1–4.4%	common snapping turtle, eastern musk turtle, painted turtle, yellow-bellied slider, spiny softshell turtle (*Apalone spinifera*) [69], Blanding’s turtle [24], eastern box turtle [70], red-eared slider [71]

^a^ 479 bp product 100% or 428 bp product 93% identical to a *Mycoplasma* sp. sequence from a red-eared slider (MG677114.1) and an eastern box turtle (KJ623620.1).

^b^ 505 bp product 97% identical to a *Mycoplasma* sp. sequence from a Blanding’s turtle (MH260079.1).

^c^ 181 bp product 100% identical to Trachemys herpesvirus 1 (MG677112.1).

^d^ Six turtles produced a 253 bp product 100% identical, one turtle produced a 272 bp product that was 99% identical, and one turtle produced a 272 bp product that was 98.5% identical to Sulawesi tortoise adenovirus (EU056826.1).

^e^ 246 bp product 100% identical to box turtle adenovirus (EU828750.1).

^f^ 277 bp product 99% identical to red-eared slider adenovirus (JX307097.1).

^g^ 257 bp product 100% identical to yellow-bellied slider adenovirus (JX307096.1).

^h^ 258 bp product 99% identical to yellow-bellied slider adenovirus (JQ801340.1).

* Names used for sequences are not officially recognized by the International Committee on Systematics of Prokaryotes or the International Committee on Taxonomy of Viruses and are only used here for descriptive purposes.

**Fig 1:**
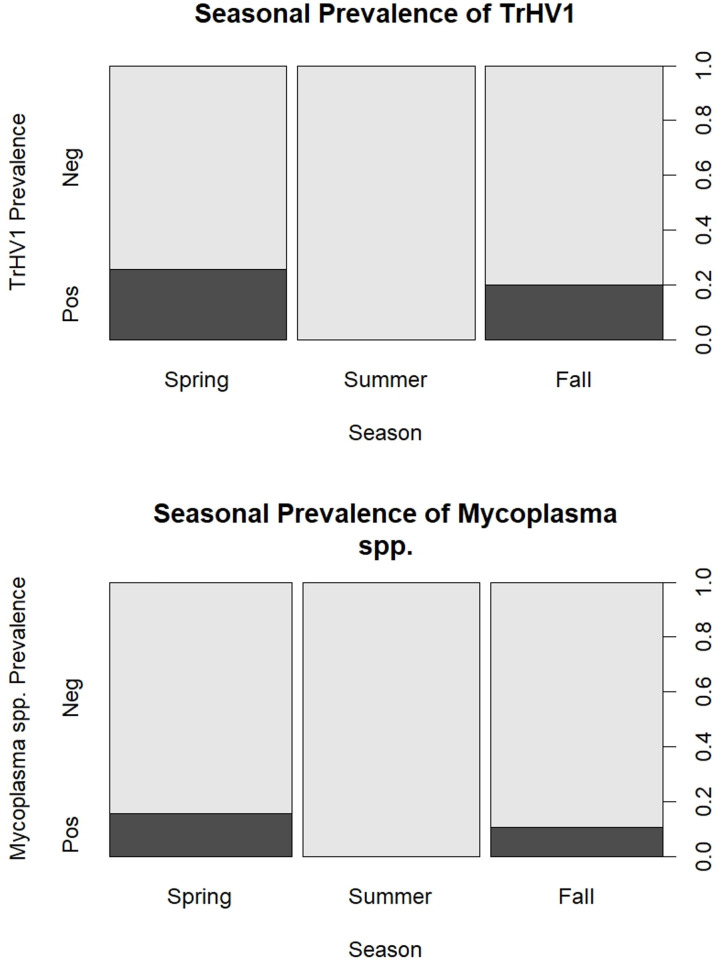
Histograms of prevalence of Trachemys herpesvirus 1 and *Mycoplasma* spp. detected in free-ranging red-eared sliders (*Trachemys scripta elegans*) in Cook County, Illinois as they vary by season.

The most parsimonious model predicting the detection of TrHV-1 included season alone (p = 0.0006) however, no contrasts were statistically significant (p > 0.05), despite TrHV-1 detections occurring only in spring (n = 9) and fall (n = 7) (Fig 1). The most parsimonious model predicting *Mycoplasma* spp. detection included the additive effects of season and location where the odds of detection were 7.9 times higher in turtles from Site A versus all other sites (p = 0.0126, 95% CI: 1.7–36.6) but contrasts for season were not statistically significant (p > 0.05). Due to the small sample size of FV3-positive animals (n = 4), statistical analysis was not performed. FV3-positive individuals were sampled in 2022 from two sites (Site A, n = 1, Site B, n = 2), and in 2021 from one site (Site C, n = 1). One was an adult male, one was an adult female, two were juvenile females, and all four turtles were sampled in fall. One turtle (adult male from Site B) was also sampled in the summer of the same year, at which time it was negative for FV3 but was detected with a 246 bp product 100% identical to box turtle adenovirus (EU828750.1). One FV3-positive adult, female had a small (2–3 mm diameter) white, mass on the gnathotheca and ulceration of the oral mucosa. No typical clinical signs of ranavirus were observed in the other adult male or two juveniles FV3-positive turtles.

Clinical signs including asymmetrical nares and appendage abnormalities along with co-infection with ectoparasite presence were not significantly associated with detection of TrHV-1 or *Mycoplasma* spp. (p > 0.05). However, 13.2% (n = 5) of turtles with leeches had at least one pathogen: TrHV-1 (n = 1), FV3 (n = 1), Emydid *Mycoplasma* sp. (n = 1), Sulawesi tortoise adenovirus (n = 1), and a co-infected turtle with TrHV-1 and Sulawesi tortoise adenovirus (n = 1). Of the 2021 and 2022 turtles that were selected for removal and necropsy (n = 42), 35.7% (n = 15, 95% CI: 23–50.8%) were detected with pathogens versus a pathogen prevalence of 27.3% (n = 46, 95% CI: 21.2–34.6%) in the turtles not selected for necropsy (n = 168); this trend was not statistically significant (p > 0.05).

### Clinical pathology models

Clinical pathology data were best predicted by varying combinations of spatiotemporal, demographic, and pathogen factors (Tables 4–6).For animals that received a CT scan, gravid females had increased total calcium (effect size = 3.5 mg/dL, p = 0.0001, 95% CI: 1.9–5.0 mg/dL) and decreased AST (effect size = 65.4 U/L, p = 0.017, 95% CI: 12.6–118.1 U/L) compared to non-gravid females. Despite females having increased total calcium, no significant differences in calcium:phosphorus ratio were observed between sexes (p > 0.05). Turtles with radiographic shell lesions had higher total solids (effect size = 1.1 g/dL, p = 0.037, 95% CI: 0.07–2.14 g/dL) and lower creatine kinase (effect size = 869.9 U/L, p = 0.002, 95% CI: 295–1344 U/L).

**Table 4:** Hematology value variability by demographic and pathogen-related factors from 185 free-ranging red-eared sliders in Cook County, IL sampled between April and October of 2021 and 2022. All reported models are significant or otherwise are stated “Null.” Bolded words are significant at least at p < 0.05. PCV = packed cell volume, TS = total solids, ESR = erythrocyte sedimentation rate, WBC = white blood cells, H:L ratio = heterophil:lymphocyte ratio.

Analyte	Year(s)	Model	Level	Model Estimate	Standard Error	Contrast	Difference	95% Confidence Interval
PCV (%)	’21-‘22	Year	2021 2022	19.9 22.4	0.751 0.661	**2022:2021**	+2.52	0.548–4.5
TS (g/dL)	’21-‘22	Year	2021 2022	4.25 3.52	0.211 0.186	**2021:2022**	+0.737	0.181–1.29
WBC/µL	’21-‘22	Year	2021 2022	16140 9252	1281 1149	**2021:2022**	+6889	3493–10284
ESR (mm)	’21-‘22	Season	Spring Summer Fall	6.72 6.93 4.36	0.374 0.374 0.489	**Spring:Fall** **Summer:Fall**	+2.356 +2.574	0.898–3.81 1.116–4.03
Heterophils/µL	’21-‘22	Year	2021 2022	1869 1019	146 131	2021:2022	+851	463–1238
Lymphocytes/µL	’21-‘22	Season	Spring Summer	3432 7912	935 919	**Summer:Spring**	+4480	1381–7579
Eosinophils/µL	’21-‘22	Season	Spring Summer Fall	930 2228 2337	228 224 213	**Fall:Spring** **Summer:Spring**	+1407 +1298	670–2144 544–2052
Basophils/µL	’21-‘22	Year	2021 2022	4883 2004	402 361	2021:2022	+2878	1812–3944
HL ratio	’21-‘22	Location	Site B Site C Site D Site A	0.242 0.831 0.170 0.389	0.046 0.113 0.142 0.037	**Site C:Site B** **Site C:Site D** **Site C:Site A**	+0.5886 +0.6613 +0.4422	0.2724–0.905 0.192–1.13066 0.134–0.75057
PCV (%)	2022	Null	–	–	–	–	–	–
TS (g/dL)	2022	Season	Spring Summer Fall	3.74 2.87 3.96	0.16 0.156 0.158	**Fall:Summer** **Fall:Spring**	+1.084 +0.87	0.557–1.61 0.338–1.4
WBC/µL	2022	Season	Spring Summer Fall	3028 9715 14815	1359 1320 1339	**Fall:Spring** **Summer:Spring** **Fall:Summer**	+11786 +6687 +5099	7245–16327 2178–11196 625–9574
ESR (mm)	2022	Season + TrHV1	Neg Pos Spring Summer Fall	6.23 4.53 5.79 6.5 3.86	0.3 0.76 0.54 0.53 0.63	**Neg:Pos** **Spring:Fall** **Summer:Fall** **Summer:Spring**	+1.7 +1.93 +2.65 +0.72	0.067–3.34 0.294–3.57 0.984–4.31 0.977–2.41
Heterophils/µL	2022	Season	Spring Summer Fall	294 1226 1509	182 176 179	**Fall:Spring** **Summer:Spring**	+1215 +932	607–1822 329–1535
Lymphocytes/µL	2022	Season	Spring Summer Fall	1680 4907 6513	823 799 811	**Fall:Spring** **Summer:Spring**	+4833 +3227	2084–7582 497–5957
Eosinophils/µL	2022	Season	Spring Summer Fall	344 1791 2990	258 251 254	**Fall:Spring** **Summer:Spring** **Fall:Summer**	+2646 +1447 +1199	1784–3509 590–2304 349–2050
Basophils/µL	2022	Season	Spring Summer Fall	614 1708 3659	355 344 349	**Fall:Spring** **Fall:Summer**	+3044 +1951	1860–4229.2 783–3117.9
HL ratio	2022	Season	Spring Fall	0.211 0.359	0.039 0.038	**Fall:Spring**	+0.147	0.0173–0.2777

**Table 5:** Plasma biochemistry value variability by demographic and pathogen-related factors from 192 free-ranging red-eared sliders in Cook County, IL sampled between April and October of 2021 and 2022. Bolded words are significant at least at p < 0.05. ME = Model Estimate, SE = Standard Error, CaP Ratio = Calcium Phosphorus Ratio, AST = aspartate transferase, GLDH = glutamate dehydrogenase.

Analyte	Year(s)	Model	Level	ME	SE	Contrast	Difference	95% Confidence Interval
Total Calcium (mg/dL)	’21-‘22	Sex + Age	Female Male Unknown Adult Juvenile	10.06 7.56 8.19 9.75 7.46	0.51 0.53 0.87 0.4 0.75	**Female:Male** **Female:Unknown** **Unknown:Male** **Adult:Juvenile**	+2.5 +1.87 +0.63 +2.29	1.51–3.48 −0.71–4.44 1.99–3.25 0.44–4.14
Phosphorus (mg/dL)	’21-‘22	Season	Spring Summer Fall	2.23 3.84 2.35	0.12 0.12 0.12	**Summer:Spring** **Summer:Fall**	+1.613 +1.487	1.227–2 1.097–1.877
Ca:Phos Ratio	’21-‘22	Season	Spring Summer Fall	4.51 2.7 4.27	0.15 0.15 0.16	**Fall:Summer** **Spring:Summer**	+1.572 +1.804	1.053–2.09 1.29–2.318
Bile Acids (µmol/L)	’21-‘22	Age	Juvenile Adult	17.41 4.97	3.15 1.00	**Juvenile:Adult**	+12.4	5.92–19
Uric Acid (mg/dL)	’21-‘22	Season	Spring Summer Fall	1.37 1.4 1.65	0.65 0.64 0.66	**Fall:Spring** **Fall:Summer**	+0.2802 +0.2564	0.0618–0.499 0.0410–0.472
Creatine Kinase (U/L)	’21-‘22	Year	2021 2022	1121 559	107 105	2021:2022	+562	267–858
AST (U/L)	’21-‘22	Age	Juvenile Adult	151 116	16.92 4.94	**Juvenile:Adult**	+35.5	0.696–70.2
Sodium (mmol/L)	’21-‘22	Season	Spring Summer Fall	133 139 137	0.72 0.89 1.01	**Summer:Spring** **Fall:Spring**	+5.97 +3.36	3.26–8.977 0.404–6.306
GLDH (U/L)	’21-‘22	Season	Spring Fall	9.9 5.29	1.27 1.23	**Spring:Fall**	+4.61	0.442–8.78
Glucose† (mg/dL)	’21-‘22	Season	Spring Summer	48 104	5.45 5.45	**Summer:Spring**	+54.7	39.8–69.6
Potassium† (mmol/L)	’21-‘22	Season	Spring Summer	3.03 3.9	0.12 0.12	**Summer:Spring**	+0.983	0.555–1.41
Total Calcium (mg/dL)	2022	Sex	Female Male	10.99 8.84	0.41 0.40	**Female:Male**	+2.155	0.797–3.51
Phosphorus (mg/dL)	2022	Season	Spring Summer Fall	2.19 4.29 2.76	0.14 0.14 0.14	**Summer:Fall** **Summer:Spring**	+1.529 +2.095	1.05–2.0047 1.62–2.5662
Ca:Phos Ratio	2022	Season	Spring Summer Fall	4.06 2.56 3.9	0.18 0.17 0.18	**Fall:Summer** **Spring:Summer**	+1.229 +1.356	0.634–1.824 0.749–1.962
Bile Acids (µmol/L)	2022	Age	Juvenile Adult	26.9 2.9	4.91 1.13	**Juvenile:Adult**	+24	14–34
Uric Acid (mg/dL)	2022	Null	–	–	–	–	–	–
Creatine Kinase (U/L)	2022	Season + Adenovirus	Neg Pos Fall Spring Summer	626 323 374 314 736	62.1 129.4 112 107 101	**Neg:Pos** **Fall:Spring** **Summer:Fall** **Summer:Spring**	+303 +60.7 +361.6 +422.4	14.8–592 −259–380.7 26.7–697 92.1–753
AST (U/L)	2022	Null	–	–	–	–	–	–
Sodium (mmol/L)	2022	Season + Myco	Neg Pos Fall Spring Summer	136 133 135 132 137	0.38 1.5 1.02 0.78 1.0	Neg:Pos Fall:Spring Summer:Fall Summer:Spring	+3.88 +3.03 +2.46 +5.49	0.78–6.98 0.74–5.32 0.32–4.6 3.25–7.74
LDH (U/L)	2022	Season	–	–	–	None significant	–	–
Glucose† (mg/dL)	2022	Season	Spring Summer	48.4 102.7	5.18 5.1	**Summer:Spring**	+54.3	39.8–68.8
Potassium† (mmol/L)	2022	Season	Spring Summer	3.12 4.01	0.16 0.15	**Summer:Spring**	+0.897	0.462–1.33

†No samples were run in Fall of 2021 or 2022. For 2021, only 67 samples were evaluated. For 2022, only 69 samples were evaluated.

**Table 6:** Plasma protein electrophoresis value variability by demographic and pathogen-related factors from 101 free-ranging red-eared sliders in Cook County, IL sampled between April and October of 2022. Bolded words are significant at least at p < 0.05.

Analyte	Model	Level	ME	SE	Contrast	Difference	95% Confidence Interval
Total Protein (g/dL)	Season	Spring Summer Fall	3.75 2.87 3.96	0.16 0.16 0.16	**Fall:Summer** **Spring:Summer**	+1.082 +0.879	0.544–1.62 0.349–1.408
Albumin:Globulin ratio	Age	Juvenile Adult	0.56 0.44	0.04 0.01	**Juvenile:Adult**	+0.126	0.0349–0.217
Prealbumin (g/L)	Location	Site B Site D Site A	0.022 0.08 0.023	0.003 0.009 0.003	**Site D:Site B** **Site D:Site A**	+0.058 +0.0573	0.0355–0.0807 0.0344–0.803
Albumin (g/L)	Season	Spring Summer Fall	1.05 0.87 1.15	0.04 0.04 0.04	**Fall:Summer** **Spring:Summer**	+0.2839 +0.1884	0.1436–0.424 0.0503–0.326
Alpha 1 globulins (g/L)	Season	Spring Summer Fall	0.2 0.16 0.23	0.01 0.01 0.01	**Fall:Summer** **Spring:Summer**	+0.0704 +0.0423	0.0325–0.1083 0.00498–0.0796
Alpha 2 globulins (g/L)	Season	Spring Summer Fall	0.74 0.38 0.6	0.03 0.03 0.03	**Spring:Fall** **Spring:Summer** **Fall:Summer**	+0.138 +0.353 +0.215	0.0327–0.244 0.250–0.4567 0.11–0.3198
Beta globulins (g/L)	Sex	–	–	–	**None significant**	–	–
Gamma globulins (g/L)	Season	Spring Summer Fall	0.9 0.63 0.95	0.05 0.05 0.05	**Fall:Summer** **Spring:Summer**	+0.3242 +0.2725	0.167–0.481 0.118–0.427

### Imaging

**Fig 2:**
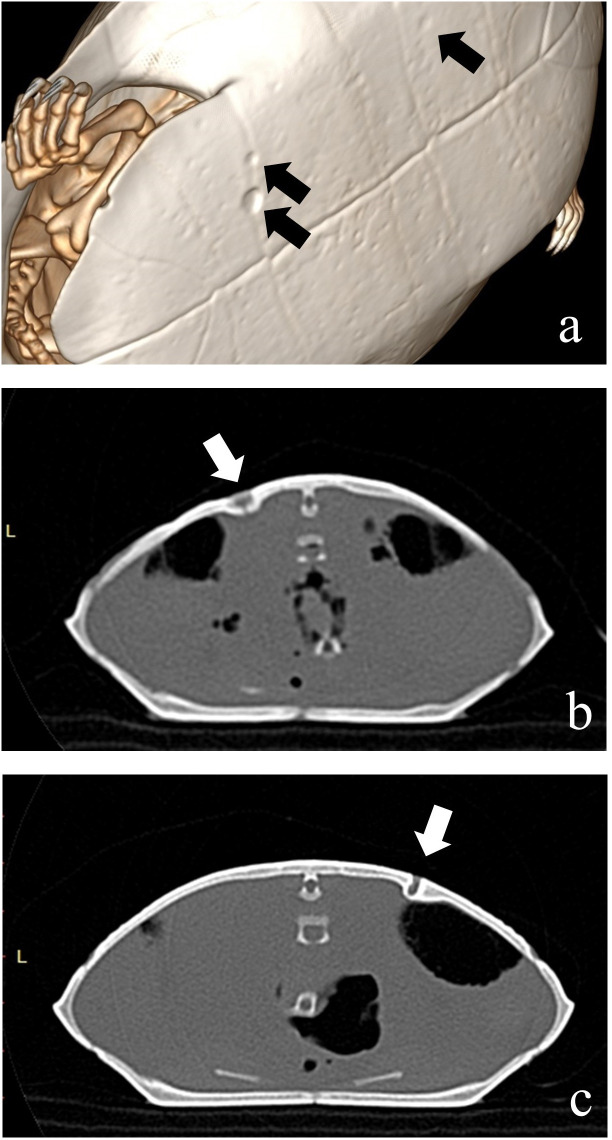
CT images of sampled red-eared sliders. **(a)** Shell erosions in the plastron of an adult, female red-eared slider (*Trachemys scripta elegans*) representative of lesions seen in 61.9% (n = 26) of sliders imaged from Cook County, IL. **(b, c)** Shell lesions composed of cystic structures encased in material of bone opacity projecting into the coelom ventrally from the carapace in two red-eared sliders. Additional post-mortem testing performed on the lesion in slider (b) revealed a focal cutaneous invagination with epidermal hyperplasia and hyperkeratosis, intralesional bacteria, fungal hyphae, and foreign debris, and dermal bone sclerosis. However, the fungal hyphae were not morphologically consistent with *Emydomyces testavorans* and both sliders tested qPCR negative on shell swabs.

In 2021 and 2022, 42 turtles were imaged via CT. More than half of the turtles (61.9%; n = 26), had radiographically-apparent erosions in skeletal plates underlying scutes (Fig 2). Two turtles (4.76%) had shell lesions composed of cystic structures encased in material of bone opacity projecting into the coelom ventrally from the carapace (Fig 2). On necropsy, one of these was described as likely an old, previous, penetrating injury. Of the females scanned (n = 28), half (50%; n = 14) had apparent ovarian follicles or shelled eggs of widely varying numbers (min = 3, max = 70, median 36.5).

### Gross and histopathology

Between 2018 and 2022, 96 turtles were necropsied with full sampling numbers available in Table 2. Turtles were necropsied from 5 locations: Site A (n = 56), Site B (n = 10), Site E (n = 10), Site G (n = 15) and Site H (n = 5). Available in Table 7 is a summary of demographic factors and additional sample numbers from necropsied turtles. On physical examinations prior to necropsy, the most common clinical abnormalities included: asymmetrical nares (n = 5), *Placobdella* spp. leech parasitism (n = 8), and shell abnormalities (n = 27) including discoloration, flaking, erosion, and pitting.

**Table 7:** A summary of the demographic information and additional samples collected from 96 necropsied, free-ranging red-eared sliders (*Trachemys scripta elegans*) from Cook County, Illinois.

	2018	2019	2020	2021	2022
Total turtles necropsied	10	24	20	21	21
Males	7	12	10	8	7
Females	3	12	9	12	13
Unknown Sex	–	–	1	1	1
Adults	10	18	17	18	19
Juveniles	–	6	3	3	2
Spring	–	–	–	7	7
Summer	10	24	10	7	7
Fall	–	–	10	7	7
Physical exam	–	–	–	7	20
Complete blood counts	–	–	–	7	19
Plasma biochemistry	–	–	–	20	19
Protein electrophoresis	–	–	–	–	19
Molecular pathogen testing	–	14	20	21	21
Computed tomography	–	–	–	21	21

**Fig 3:**
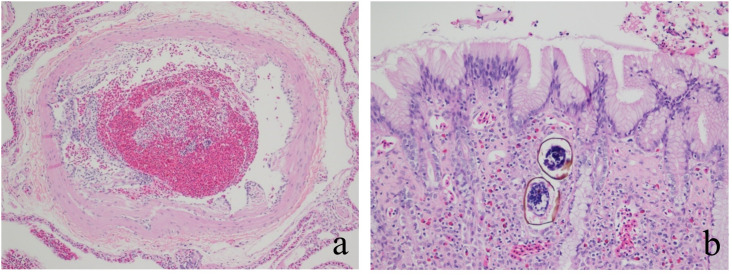
Representative histologic changes associated with Spirorchiidae infestation in red-eared sliders (*Trachemys scripta elegans*) from Cook County, IL. **(a)** Pulmonary arterial endarteritis is characterized by subintimal, short villous projections of fibrous tissue that protrude into the lumen. Changes are indicative of previous insult/irritation associated with intravascular parasitism. **(b)** Gastric lamina propria contains two metazoan ova with thin, brown-pigmented, refractile walls consistent with trematodes (Spirorchiidae, presumed).

Across years almost all turtles (97.9%; n = 94) were diagnosed with at least one parasite: 71.9% (n = 69) had gastrointestinal nematodes morphologically consistent with the order Spirurida, 51.0% (n = 49) had trematodes (suspected Spirorchiidae, Fig 3) in a wide variety of organs, 42.7% (n = 41) had acanthocephalan parasites mostly in the intestines, 18.8% (n = 18) had unspecified metazoan parasites in the stomach, pancreas, or intestines, 10.4% (n = 10) had renal myxosporidiosis (suspected *Myxidium scripta*), 2.1% (n = 2) had external leeches, and 1% (n = 1) had suspected external mites.

Over half (57.3%; n = 55) of all turtles were diagnosed with at least one vascular condition: 50.0% (n = 48) had endarteritis and 19.8% (n = 19) had medial smooth muscle hyperplasia. Other notable conditions included: yolk coelomitis (n = 2), ovarian follicular degeneration (n = 5), small intestinal lymphosarcoma (n = 1), right hindlimb amputations (n = 4), kyphosis (n = 1), and scoliosis (n = 1).

Forty-two of the 96 necropsied turtles were also tested for pathogens and 15 were positive for at least one pathogen. However, there were no gross or histologic lesions that could be associated with molecularly-detected pathogens.

### Reference intervals

After exclusion criteria were applied to the 210 individuals sampled in 2021 and 2022, 96 turtles remained and were used to create reference intervals for clinical pathology testing (Table 8). Final demographics of included samples were as follows: age: 90 adults, 6 juveniles; sex: 41 females, 48 males, 7 turtles of unknown sex; season: 39 fall samples, 39 spring samples, and 18 summer samples; year: 41 2021 samples, 55 2022 samples. Plasma protein electrophoresis was only performed in 2022 and 54 turtles were included in the reference intervals.

**Table 8:** Hematology, plasma biochemistry, and plasma electrophoresis 95% reference intervals created from up to 96 samples from red-eared sliders (*Trachemys scripta elegans*). PCV = packed cell volume, TS = total solids, ESR = erythrocyte sedimentation rate, WBC = white blood cells, H:L ratio = heterophil:lymphocyte ratio, CaP Ratio = Calcium Phosphorus Ratio, AST = aspartate transferase, GLDH = glutamate dehydrogenase.

Analyte	N	Mean	SD	Med	Min	Max	Distribution	Method used	Ref Int	90% CI LB	90% CI HB
PCV (%)	96	21.5	6.2	22.3	4.5	34.5	Normal	Parametric	9.3–33.8	7.5–11.0	32.0–35.5
TS (g/dL)	95	3.7	1.0	3.8	1.4	6.6	Normal	Parametric	1.7–5.7	1.5–2.0	5.4–6.0
ESR (mm)	76	6.06	3.03	5.47	1.00	20.90	Non-normal	Robust	0.52–10.56	−0.41–1.52	9.48–11.74
WBC/µL	96	11760	8748	10909	93	45056	Non-normal	Robust	0–27938	−10449 - −4141	24645–31557
Heterophils/µL	96	1688	1517	1205	6	7430	Non-normal	Robust	0–4571	−2392 - −1191.22	4009–5269
Lymphocytes/µL	95	4873	4790	3300	312	27045	Non-normal	Robust	0–13495	−9168 - -4298^b^	11213 – 16092^b^
Monocytes/µL	96	294	422	136	0	3112	Non-normal	Robust	0–1044	−1071 - -437^b^	718 – 1372^b^
Eosinophils/µL	96	1733	1540	1290	0	8208	Non-normal	Robust	0–4659	−2358 - −1125	4084 – 5388^b^
Basophils/µL	95	3041	2658	3034	19	18670	Non-normal	Robust	0–8040	−4034 - -830^b^	6323 – 9610^b^
H:L ratio	94	0.47	0.48	0.33	0.06	2.80	Non-normal	Robust	0–1.31	−0.99 - −0.38^b^	1.02 – 1.65^b^
Total Ca2+ (mg/dL)	88	9.5	2.6	9.1	5.3	17.9	Non-normal	Robust	3.7–14.2	2.8–4.5	13.1–15.3
Phosphorus (mg/dL)	89	2.6	1.2	2.4	1.0	6.0	Non-normal	Robust	0.1–4.7	−0.3–0.5	4.2–5.1
CaP Ratio	87	4.15	1.57	4.16	1.63	9.10	Non-normal	Robust	0.78–7.05	0.22–1.36	6.48–7.68
Bile Acids (µmol/L)	56	2.1	0.12	2.0	2.0	2.8	Non-normal	Parametric	1.7–2.4	1.7–1.8	2.4–2.5
Uric Acid (mg/dL)	89	1.6	0.5	1.5	1.2	4.4	Non-normal	Parametric	0.6–2.5	0.5–0.8	2.4–2.7
Creatine Kinase (U/L)	84	777	729	522	10	3314	Non-normal	Robust	0–2070	−1337 - -671^b^	1726 – 2440^b^
AST (U/L)	87	114	50	105	27	266	Non-normal	Robust	8–210	−6–22	191–230
Sodium (mmol/L)	67	137	4	136	129	146	Normal	Robust	129–145	128–131	143–146
GLDH (U/L)	86	5.9	4.1	4.5	0.2	32.0	Non-normal	Robust	0–17.7	−6.1 - −0.5^b^	14.8 – 20.8^b^
Glucose (mg/dL)	31	81	47	65	27	195	Non-normal	Robust	0–169	−67 - -10^b^	134 – 202^b^
Potassium (mmol/L)	31	3.6	0.84	3.5	2.4	5.9	Normal	Robust	1.9–5.3	1.5 – 2.4^b^	4.8 – 5.7^b^
Total protein (g/dL)	53	3.6	1.1	3.6	1.4	6.6	Normal	Parametric	1.5–5.7	1.1–2.0	5.3–6.1
Albumin/Globulin	53	0.45	0.1	0.45	0.25	0.65	Normal	Parametric	0.24–0.63	0.2–0.28	0.59–0.67
Pre-albumin (g/L)	52	0.02	0.01	0.02	0	0.06	Non-normal	Robust	−0.02–0.05	−0.03 – −0.01	0.04 – 0.05^b^
Pre-albumin (%)	47	0.5	0.45.	0.5	0	3.3	Non-normal	Robust	0.11–0.88	0.03 – 0.21^b^	0.8 – 0.96^b^
Albumin (g/L)	54	1.05	0.27	1.06	0.29	1.67	Normal	Parametric	0.52–1.58	0.41–0.62	1.47–1.68
Albumin (%)	54	29.12	5.18	29.6	18.1	39.1	Normal	Parametric	18.96–39.28	16.98–20.95	37.3–41.27
Alpha 1 globulins (g/L)	53	0.20	0.08	0.18	0.1	0.45	Non-normal	Robust	0.02–0.35	−0.02 – 0.06^b^	0.31 – 0.4^b^
Alpha 1 globulins (%)	51	5.33	0.9	5.3	3.8	7.5	Normal	Parametric	3.56–7.1	3.2 – 3.92^b^	6.75 – 7.46^b^
Alpha 2 globulins (g/L)	53	0.55	0.2	0.52	0.2	1.08	Non-normal	Robust	0.11–0.95	0.03–0.18	0.85 – 1.04^b^
Alpha 2 globulins (%)	54	15.37	3.7	15	9.5	25.1	Non-normal	Robust	7.3–22.6	6.0–8.7	20.9 – 24.3^b^
Beta globulins (g/L)	54	0.97	0.39	0.92	0.33	2.04	Normal	Parametric	0.2–1.74	0.05–0.35	1.59–1.89
Beta globulins (%)	54	26	5.3	25.3	14.5	39.5	Normal	Parametric	15.7–36.4	13.7–17.7	34.4–38.4
Gamma globulins (g/L)	51	0.85	0.28	0.81	0.34	1.68	Non-normal	Robust	0.24–1.41	0.12 – 0.36^b^	1.29 – 1.54^b^
Gamma globulins (%)	54	23.4	4.1	24	12.4	29.9	Normal	Parametric	15.4–31.4	13.9–17	29.8–32.9

^a^ Outliers removed: TS (7.7), ESR (1.0, 20.9), lymphocytes (55), basophils (20275), H:L ratio (0.044, 0.048), total CA2+ (4.8), phosphorus (7.2), Ca:P ratio (1.4, 11.0), bile acids (1.2, 1.0, 1.1, 1.3, 3.0, 3.9, 5.1, 4.5, 3.0, 3.9, 7.2, 3.0, 1.3, 4.4), AST (21, 394, 419), Sodium (154, 117), GLDH (1, 74, 87), total protein (7.7), pre-albumin (0.09, 0.15), pre-albumin % (1.7, 3.3, 3.4, 2, 1.3, 1.5, 1.4), Alpha 1 globulins (0.06), Gamma globulins (0.23, 1.96, 2.09)

^b^ Denotes confidence intervals that exceed 0.2 times the width of the reference interval which may be broader than recommended by Friedrichs et al., 2012

## Discussion

The use of free-ranging red-eared sliders as sentinels for disease may inform pathogen risk assessments for co-occurring species of conservation concern. Health assessments performed on free-ranging RES in Cook County between 2018 and 2022 resulted in the detection of multiple pathogens including FV3, TrHV-1, adenoviruses [72], *Mycoplasma* sp., human-pathogenic *Leptospira* spp., *Emydomyces testavorans*, and *Salmonella typhimurium*. Notably, these pathogens were present in the absence of any gross or histologic lesions indicative of associated disease. Common necropsy findings were reflective of incidental ecto- and endoparasitism unrelated to pathogens of concern. Reference intervals were created for hematology, plasma biochemistry, and plasma protein electrophoresis. The results of this study provide baseline health information for free-ranging, non-native RES, demonstrate the utility of molecular diagnostics in free-ranging wildlife health assessments, and provide evidence of the disease threats that invasive RES may be harboring within native turtle populations.

It is unsurprising that the detection of multiple pathogens (*Mycoplasma* spp., TrHV-1, and FV3) was associated with sample season as temporal effects have previously been reported for chelonian pathogens. This may reflect the difference in optimal viral shedding conditions based on season, host species, or location-based environmental characteristics for different pathogens [27,41,73,74]. FV3 was detected in four turtles, with all detections occurring in fall over different years and sites. Previous research generally reports ranavirus outbreaks during the active turtle season (spring) with infected red-eared slider adults having shorter survival times in cooler temperatures and infected red-eared slider juveniles having shorter survival times than adults [27,73,74]. Although sampling season was associated with *Mycoplasma* spp. and TrHV-1 detection, statistical comparisons between specific seasons were not significant. In the future, increasing the sample sizes for each season may allow for the detection of specific statistically-significant seasonal contrasts. Previously, *Mycoplasma* spp. detection has increased, in separate studies, in fall or in warmer months in tortoises which may be related to optimal growth conditions for the organism [75,76]. Herpesvirus detection appears to have variable seasonal associations depending on the virus and host with *Terrapene* herpesvirus 1 in Eastern box turtles (*Terrapene carolina carolina*) detected more in summer or fall and *Emydoidea* herpesvirus 1 in Blanding’s turtles detected more in spring or fall [24,41,77]. In free-ranging reptiles, a variety of factors may lead to seasonal detection of pathogens including recrudescence of latent infections during times of physiologic stress (i.e., breeding or nesting), increased conspecific interaction during specific seasons, environmental factors that increase pathogen survival outside the host, and immunosuppression in late winter-early spring in species that undergo brumation [24,77–80]. Despite a lack of statistically significant seasonal associations, detection of TrHV-1 only occurred in spring and fall sampling seasons and may be shed more during recovery from brumation (spring) and metabolically preparing for brumation (fall). The nesting season for RES in Illinois can be long and variable (April – July) but the physiologic stress associated with preparing for nesting might also increase shedding of this virus in spring [81]. Knowing in which season a pathogen is most likely to be shed can help guide when specific disease surveillance, prevention, and/or management strategies are most likely to be effective. Further longitudinal study and sampling during varied seasons may help elucidate seasonal factors related to pathogen detection.

The FV3 positive turtles in the present study may represent persistently-infected turtles that were shedding due to immunosuppression related to season or temperature, turtles with a recent infection just prior to or post-display of clinical signs, or environmental contamination. In fact, a lack of association of any clinical signs with any pathogen are not unexpected as a recent literature review of turtle health studies determined that 70 of 275 antemortem health surveys revealed no abnormal clinical signs in studied populations [82]. This may suggest many turtles harbor subclinical infections, the clinical period of infection can be rapid and missed by health surveys, or that infections may lead more often to mortality missed by surveys of living animals. Despite the lack of statistical association, the finding of pathogens being detected in several turtles also observed with leeches may be worthy of further investigation. Recently, a study in green sea turtles (*Chelonia mydas*) found a possible association between marine leech parasitism and chelonid herpesvirus 5 infection [83]. Further analysis or a larger sample size may help elucidate whether any such relationship exists in freshwater leeches and turtles.

One site, Site A, was associated with significant pathogen detection. Site A is a relatively isolated marshland with remnant seepage water qualities abutting a large housing subdivision and supporting a variety of amphibians and other chelonians such as Blanding’s turtles, common snapping turtles (*Chelydra serpentina*), painted turtles (*Chrysemys picta*), and Eastern musk turtles (*Sternotherus odoratus*). The increased prevalence of *Mycoplasma* spp. at Site A compared to other sites may be due to multiple factors. In other chelonians, *Mycoplasma* spp., transmission is hypothesized to be direct and so increased prevalence may be facilitated by increased conspecific and inter-species interaction [84] Site A and Site G are the only sites that do not have a river within 1000 feet of the site. Therefore, Site A might represent a more closed population of RES compared to most other sites. A relatively more confined population of turtles may experience more nose-to-nose interaction, thereby leading to a higher prevalence of pathogens like *Mycoplasma* spp. Additionally, many sites are accessible by members of the public with Site A being adjacent to a large subdivision. It is possible that RES kept as pets are illegally released into either site as is known to occur worldwide [7]. Recently, studies have demonstrated host-switching of endoparasites from invasive RES to native turtles in Europe [11,85]. It is possible that pathogens detected in these locations are a product of higher population densities at this site or higher chelonian biodiversity at this site, but additional study would be needed to investigate this link.

Boney lesions in the carapace and plastron were commonly identified via CT scan and may be due to bacterial infection as described in septicemic cutaneous ulcerative disease, previous trauma, nutritional disease, algae invasion of the shell, or fungal infection with agents like *Emydomyces testavorans* [68,86–89]. Two turtles had intracoelomic cysts that appeared as expansile lesions extending from the coelomic surface of the shell with radiolucent interior cavities. Imaging characteristics of these lesions were similar to epithelial inclusion cysts described in association with *E. testavorans* infection in a variety of other turtle species [68]. Shell swabs of these turtles failed to detect *E. testavorans* and there was no histologic evidence of fungal infection which may reflect that these lesions were not due to the organism, the infection was already resolved, or that the fungus was deep within the lesion beyond the reach of the swab. This demonstrates the potential value of incorporating CT into disease surveillance programs to either increase or allay suspicion for the presence of pathogens like *E. testavorans*.

Multiple hematology, biochemistry, and protein electrophoresis values were significantly associated with season, site, or pathogen detection. Differences in total WBC counts, heterophils, lymphocytes, and eosinophils, with spring typically having the lowest counts and values increasing during summer and fall, provides increased resolution to previously observed seasonal WBC differences in yellow-bellied sliders where heterophils were higher in summer and basophils higher in winter [90]. These seasonal differences are likely due to changes in normal physiologic processes such as emergence from brumation in spring. Plasma biochemistry values also exhibited season specific changes except for calcium, in which adult females had classically higher levels likely related to reproductive needs for egg production.

On necropsy, an extremely high percentage (97.7%) of RES were diagnosed with at least one parasite. The high number of nematodes that were morphologically compatible with spirurids was consistent with previous reports in Emydidae and may represent an enzootic pathogen in these species [91,92]. Variable numbers of trematode ova were disseminated throughout multiple tissues, and rare intravascular adult trematodes were detected. Infection with Spirorchiidae was presumed, and secondary vascular lesions (i.e., proliferative endarteritis and medial smooth muscle hyperplasia) were reactive changes consequent to intravascular parasitism [93]. Interestingly, on necropsy and histopathology, no classic enteritis, hepatitis, or splenitis was observed in turtles positive for adenoviruses and no classic abnormal upper respiratory tract lesions were observed in turtles detected with *Mycoplasma* spp., FV3, and/or TrHV-1 [94–97]. The lack of gross or histopathologic lesions associated with pathogen detection may indicate that although these pathogens were being shed, they were not causing disease in infected turtles at the time of detection. Absence of associated disease could indicate that infected animals were carriers, RES may not be susceptible to disease caused by these pathogens and/or the appropriate environmental conditions necessary to cause disease may not have been present. Finally, previous studies have also inoculated turtles with known pathogens, like FV3, and detected no or only mild signs of disease on gross and histopathology suggesting that disease is likely multifactorial and temporal. Timing of RES necropsies in the current study may have failed to appropriately capture time course of disease [98].

The reference intervals generated by this study, to the author’s knowledge, are the second published intervals created from free-ranging RES using ASVCP guidelines. Compared to invasive red-eared sliders sampled in September in Germany, PCV was relatively similar while leukocyte counts differed significantly in this study [31]. This present study found approximately five times greater total leukocyte counts, two times the heterophil count, 15 times the basophil count, five times the lymphocyte count, and about half of the heterophil:lymphocyte ratio. These comparisons likely demonstrate geographical, seasonal, and health-related differences between these two populations. Compared to captive RES, this study found that total leukocytes were only almost two times higher, basophils were three times higher, and heterophils in free-ranging RES in this study were about half of the amount observed in captive RES [29]. While broadly, supplied reference intervals can be used for general interpretation of red-eared slider hematology, these differences further highlight how these intervals can be highly species, location, environmental, and population-specific.

Possible limitations of this study include altered hematology results due to risk of lymph contamination by using the subcarapacial sinus as the venipuncture site, limited sampling of juvenile turtles which can interfere with the ability to make epidemiologic inferences about the population, and the sampling of turtles primarily caught in traps as only those turtles healthy enough to make it into the trap will be sampled. This study was subject to selection bias for the turtles sent for necropsy and histopathology as selection was mostly based on the presence of abnormal clinical signs. Clinical abnormality prevalence in those animals may be elevated compared to a normal, free-ranging RES population. Only a limited number of turtles were sampled during each sampling period possibly underpowering the ability to detect pathogens at a low prevalence. Differences in hematology analytes existed between years and so future researchers may need to create annual or seasonal population hematology reference intervals. In this study, samples sizes per season were too small to create season-specific reference ranges. Additionally, sample size limitations occurred for several analytes resulting in larger than desirable WCI/ WRI (WCI: confidence interval width, WRI: reference interval width) for reference intervals [56]. Increasing the sample size in future studies would improve the resolution of reference interval boundaries. Additionally, phylogenetic analysis should be performed in the future to examine relationships between newly discovered pathogens.

RES were detected with several pathogens that have previously been detected in Blanding’s turtles including *Salmonella typhimurium*, a human-pathogenic *Leptospira* sp., and a Blanding’s turtle *Mycoplasma* sp. [24]. These shared pathogens suggest the potential for transmission to occur between these species and either produce associated morbidity and mortality or contribute to the formation of a larger pathogen reservoir. Also, the finding of FV3 in RES in Cook County, at a site shared by endangered turtle species like the Blanding’s turtle, is cause for concern as this virus has been associated with severe disease and mass mortality in a variety of tortoises, emydid turtles, amphibians, and fish [27,65]. If Blanding’s turtles are susceptible to FV3 infection, interaction with shedding carrier RES may lead to disease or even mortality in this fragile species.

The continued spread of invasive RES represents a multi-pronged threat to native chelonians via direct competition and the introduction and maintenance of pathogens within the environment. Exposure of new chelonian populations to TrHV-1, adenoviruses, frog virus 3, *Mycoplasma* spp., ecto- and endo-parasites, and other disease agents may increase if RES are allowed to disperse or are continuously released into new areas. Future studies are needed to determined the type and extent of the threat to native turtle health that RES may play. However, if sufficient threat is determined, possible management options include RES removal from non-native ranges, environmental manipulation to discourage or prevent their spread, and preventative medicine strategies like vaccination of native reptiles for RES-carried pathogens that may be available in the future. Additionally, public education and increasing awareness of the potential effects of these non-native turtles in the region remain vital for attempting to curb any future releases of this species. Continued surveillance of this species will be increasingly important as wildlife managers determine methods for chelonian population disease risk management.

## Supporting information

S1 File AIC tables and contrasts. Akaike’s information criterion (AIC) tables and contrasts for all tested clinical pathology parameters.
(DOCX)

S2 File Master slider working sheet for PLOS. Complete dataset for red-eared sliders sampled in Cook County, IL between 2018 and 2022.
(XLSX)
