# Supplementary material for: Health assessment of non-native red-eared sliders (Trachemys scripta elegans) and their impact potential on native species
Source: PLoS One. 2025 Oct 6;20(10):e0333786. doi: 10.1371/journal.pone.0333786 (PMC12500119; doi:10.1371/journal.pone.0333786)
Supplement: S1 File — Akaike’s information criterion (AIC) tables and contrasts for all tested clinical pathology parameters. (DOCX) [file pone.0333786.s001.docx]

AIC Tables

### 21-22 PCV

Model selection based on AIC:

K AIC Delta_AIC AICWt Cum.Wt LL

Year 3 1205.07 0.00 0.61 0.61 -599.54

Location 5 1208.06 2.99 0.14 0.74 -599.03

null 2 1209.38 4.31 0.07 0.81 -602.69

Myco 3 1209.64 4.57 0.06 0.87 -601.82

SeaMyco 5 1210.35 5.28 0.04 0.92 -600.18

Sea 4 1210.41 5.34 0.04 0.96 -601.21

Age 3 1211.38 6.31 0.03 0.98 -602.69

Sex 4 1212.33 7.26 0.02 1.00 -602.17

contrast estimate SE df t.ratio p.value

Year2021 - Year2022 -2.52 1 179 -2.520 0.0126

### 21-22 TS

Model selection based on AIC:

K AIC Delta_AIC AICWt Cum.Wt LL

Year 3 745.99 0.00 0.50 0.50 -370.00

SeaMyco 5 747.84 1.85 0.20 0.69 -368.92

Sea 4 748.83 2.84 0.12 0.81 -370.41

Age 3 750.36 4.37 0.06 0.87 -372.18

null 2 750.78 4.79 0.05 0.91 -373.39

Sex 4 750.85 4.86 0.04 0.96 -371.42

Myco 3 751.15 5.16 0.04 1.00 -372.58

Location 5 755.82 9.83 0.00 1.00 -372.91

contrast estimate SE df t.ratio p.value

Year2021 - Year2022 0.737 0.282 179 2.616 0.0096

### 21-22 WBC

K AIC Delta_AIC AICWt Cum.Wt LL

Year 3 3970.22 0.00 0.98 0.98 -1982.11

Sea 4 3979.50 9.27 0.01 0.99 -1985.75

SeaMyco 5 3980.14 9.92 0.01 1.00 -1985.07

Myco 3 3983.07 12.84 0.00 1.00 -1988.53

Age 3 3983.44 13.21 0.00 1.00 -1988.72

null 2 3983.75 13.53 0.00 1.00 -1989.88

Sex 4 3985.56 15.34 0.00 1.00 -1988.78

Location 5 3986.93 16.71 0.00 1.00 -1988.46

contrast estimate SE df t.ratio p.value

Year2021 - Year2022 6889 1721 182 4.003 0.0001

### 21-22 ESR

Model selection based on AIC:

K AIC Delta_AIC AICWt Cum.Wt LL

Sea 4 745.02 0.00 0.72 0.72 -368.51

SeaMyco 5 746.96 1.94 0.27 1.00 -368.48

Year 3 759.32 14.30 0.00 1.00 -376.66

null 2 759.99 14.97 0.00 1.00 -377.99

Myco 3 761.94 16.93 0.00 1.00 -377.97

Age 3 761.98 16.97 0.00 1.00 -377.99

Location 4 763.54 18.53 0.00 1.00 -377.77

Sex 4 763.84 18.83 0.00 1.00 -377.92

contrast estimate SE df t.ratio p.value

Fall - Spring -2.356 0.616 147 -3.826 0.0006

Fall - Summer -2.574 0.616 147 -4.179 0.0001

Spring - Summer -0.218 0.530 147 -0.411 0.9112

### 21-22 Heterophils

Model selection based on AIC:

K AIC Delta_AIC AICWt Cum.Wt LL

Year 3 3171.33 0.00 0.99 0.99 -1582.67

Location 5 3180.34 9.00 0.01 1.00 -1585.17

Myco 3 3186.53 15.19 0.00 1.00 -1590.26

null 2 3187.40 16.07 0.00 1.00 -1591.70

Age 3 3189.12 17.79 0.00 1.00 -1591.56

Sex 4 3189.35 18.02 0.00 1.00 -1590.68

SeaMyco 5 3190.34 19.01 0.00 1.00 -1590.17

Sea 4 3191.06 19.73 0.00 1.00 -1591.53

contrast estimate SE df t.ratio p.value

Year2021 - Year2022 851 196 182 4.333 <.0001

### 21-22 Lymphocytes

Model selection based on AIC:

K AIC Delta_AIC AICWt Cum.Wt LL

Sea 4 3791.61 0.00 0.61 0.61 -1891.81

SeaMyco 5 3793.23 1.62 0.27 0.88 -1891.62

Year 3 3795.81 4.20 0.08 0.96 -1894.90

null 2 3799.33 7.72 0.01 0.97 -1897.66

Myco 3 3799.66 8.05 0.01 0.98 -1896.83

Age 3 3800.04 8.43 0.01 0.99 -1897.02

Sex 4 3800.84 9.23 0.01 1.00 -1896.42

Location 5 3803.95 12.34 0.00 1.00 -1896.97

contrast estimate SE df t.ratio p.value

Fall - Spring 1754 1282 181 1.368 0.3597

Fall - Summer -2726 1270 181 -2.146 0.0836

Spring - Summer -4480 1311 181 -3.416 0.0023

### 21-22 Eosinophils

Model selection based on AIC:

K AIC Delta_AIC AICWt Cum.Wt LL

SeaMyco 5 3271.44 0.00 0.52 0.52 -1630.72

Sea 4 3271.68 0.24 0.47 0.99 -1631.84

Sex 4 3279.40 7.96 0.01 1.00 -1635.70

Myco 3 3289.03 17.59 0.00 1.00 -1641.52

Age 3 3290.09 18.65 0.00 1.00 -1642.04

null 2 3290.94 19.50 0.00 1.00 -1643.47

Year 3 3291.70 20.26 0.00 1.00 -1642.85

Location 5 3291.80 20.36 0.00 1.00 -1640.90

contrast estimate SE df t.ratio p.value

Fall - Spring 1407 312 181 4.510 <.0001

Fall - Summer 109 309 181 0.352 0.9338

Spring - Summer -1298 319 181 -4.066 0.0002

### 21-22 Basophils

Model selection based on AIC:

K AIC Delta_AIC AICWt Cum.Wt LL

Year 3 3543.95 0.00 1 1 -1768.98

Location 5 3565.49 21.54 0 1 -1777.75

Age 3 3568.25 24.30 0 1 -1781.13

null 2 3568.61 24.66 0 1 -1782.31

Myco 3 3569.33 25.38 0 1 -1781.67

Sea 4 3570.62 26.67 0 1 -1781.31

SeaMyco 5 3571.30 27.35 0 1 -1780.65

Sex 4 3571.88 27.93 0 1 -1781.94

contrast estimate SE df t.ratio p.value

Year2021 - Year2022 2878 540 182 5.327 <.0001

### 21-22 HL ratio

Model selection based on AIC:

K AIC Delta_AIC AICWt Cum.Wt LL

Location 5 166.58 0.00 0.99 0.99 -78.29

Year 3 177.50 10.92 0.00 1.00 -85.75

Sea 4 180.71 14.13 0.00 1.00 -86.35

Sex 4 181.33 14.75 0.00 1.00 -86.67

SeaMyco 5 182.14 15.56 0.00 1.00 -86.07

null 2 185.65 19.07 0.00 1.00 -90.83

Age 3 187.20 20.62 0.00 1.00 -90.60

Myco 3 187.64 21.06 0.00 1.00 -90.82

contrast estimate SE df t.ratio p.value

Burnham - Flatwoods -0.5886 0.1219 180 -4.827 <.0001

Burnham - Park Ridge 0.0727 0.1488 180 0.488 0.9616

Burnham - Powderhorn -0.1464 0.0594 180 -2.466 0.0689

Flatwoods - Park Ridge 0.6613 0.1810 180 3.653 0.0019

Flatwoods - Powderhorn 0.4422 0.1189 180 3.718 0.0015

Park Ridge - Powderhorn -0.2191 0.1464 180 -1.497 0.4416

### 21-22 Total calcium

Model selection based on AIC:

K AIC Delta_AIC AICWt Cum.Wt LL

SexAge 5 939.76 0.00 0.88 0.88 -464.88

Sex 4 943.75 3.99 0.12 1.00 -467.87

Age 3 969.64 29.89 0.00 1.00 -481.82

null 2 975.30 35.54 0.00 1.00 -485.65

Sea 4 976.07 36.31 0.00 1.00 -484.04

Myco 3 976.30 36.55 0.00 1.00 -485.15

Year 3 976.38 36.62 0.00 1.00 -485.19

SeaMyco 5 977.73 37.97 0.00 1.00 -483.86

Location 5 978.36 38.61 0.00 1.00 -484.18

LocMyco 6 979.34 39.58 0.00 1.00 -483.67

contrast estimate SE df t.ratio p.value

Female Adult - Male Adult 2.495 0.418 187 5.970 <.0001

Female Adult - Unknown Adult 1.866 1.091 187 1.709 0.5273

Female Adult - Female Juvenile 2.288 0.938 187 2.441 0.1478

Female Adult - Male Juvenile 4.783 1.058 187 4.519 0.0002

Female Adult - Unknown Juvenile 4.154 0.946 187 4.391 0.0003

Male Adult - Unknown Adult -0.629 1.108 187 -0.568 0.9930

Male Adult - Female Juvenile -0.206 0.994 187 -0.208 0.9999

Male Adult - Male Juvenile 2.288 0.938 187 2.441 0.1478

Male Adult - Unknown Juvenile 1.660 0.930 187 1.785 0.4781

Unknown Adult - Female Juvenile 0.423 1.802 187 0.235 0.9999

Unknown Adult - Male Juvenile 2.917 1.830 187 1.594 0.6033

Unknown Adult - Unknown Juvenile 2.288 0.938 187 2.441 0.1478

Female Juvenile - Male Juvenile 2.495 0.418 187 5.970 <.0001

Female Juvenile - Unknown Juvenile 1.866 1.091 187 1.709 0.5273

Male Juvenile - Unknown Juvenile -0.629 1.108 187 -0.568 0.9930

### 21-22 Phosphorus

Model selection based on AIC:

K AIC Delta_AIC AICWt Cum.Wt LL

Sea 4 519.24 0.00 0.7 0.7 -255.62

SeaMyco 5 520.97 1.72 0.3 1.0 -255.48

Sex 4 585.61 66.37 0.0 1.0 -288.81

Year 3 598.10 78.86 0.0 1.0 -296.05

LocMyco 6 600.96 81.72 0.0 1.0 -294.48

Location 5 605.20 85.96 0.0 1.0 -297.60

Myco 3 605.41 86.17 0.0 1.0 -299.71

null 2 609.46 90.22 0.0 1.0 -302.73

Age 3 611.31 92.06 0.0 1.0 -302.65

contrast estimate SE df t.ratio p.value

Fall - Spring 0.127 0.166 188 0.765 0.7251

Fall - Summer -1.487 0.165 188 -9.006 <.0001

Spring - Summer -1.613 0.164 188 -9.853 <.0001

### 21-22 CaP ratio

Model selection based on AIC:

K AIC Delta_AIC AICWt Cum.Wt LL

Sea 4 627.85 0.00 0.7 0.7 -309.92

SeaMyco 5 629.57 1.72 0.3 1.0 -309.78

Year 3 683.06 55.22 0.0 1.0 -338.53

Age 3 687.16 59.31 0.0 1.0 -340.58

Sex 4 689.20 61.35 0.0 1.0 -340.60

Location 5 690.18 62.34 0.0 1.0 -340.09

LocMyco 6 690.98 63.14 0.0 1.0 -339.49

null 2 692.49 64.64 0.0 1.0 -344.24

Myco 3 692.54 64.69 0.0 1.0 -343.27

contrast estimate SE df t.ratio p.value

Fall - Spring -0.233 0.220 188 -1.057 0.5423

Fall - Summer 1.572 0.219 188 7.165 <.0001

Spring - Summer 1.804 0.218 188 8.292 <.0001

### 21-22 Bile acids

Model selection based on AIC:

K AIC Delta_AIC AICWt Cum.Wt LL

Age 3 1297.34 0.00 0.83 0.83 -645.67

Sex 4 1303.07 5.73 0.05 0.88 -647.54

Sea 4 1303.63 6.29 0.04 0.91 -647.81

Location 5 1303.82 6.48 0.03 0.95 -646.91

Year 3 1304.87 7.53 0.02 0.97 -649.44

LocMyco 6 1305.16 7.82 0.02 0.98 -646.58

SeaMyco 5 1305.55 8.21 0.01 1.00 -647.77

null 2 1309.13 11.79 0.00 1.00 -652.56

Myco 3 1311.13 13.79 0.00 1.00 -652.56

contrast estimate SE df t.ratio p.value

Adult - Juvenile -12.4 3.3 163 -3.769 0.0002

### 21-22 Uric acid

Model selection based on AIC:

K AIC Delta_AIC AICWt Cum.Wt LL

Sea 4 293.29 0.00 0.62 0.62 -142.64

SeaMyco 5 295.04 1.75 0.26 0.88 -142.52

Location 5 298.85 5.56 0.04 0.92 -144.42

Age 3 300.14 6.86 0.02 0.94 -147.07

null 2 300.50 7.22 0.02 0.96 -148.25

Year 3 300.52 7.23 0.02 0.97 -147.26

LocMyco 6 300.76 7.47 0.01 0.99 -144.38

Myco 3 302.35 9.06 0.01 1.00 -148.18

Sex 4 303.20 9.92 0.00 1.00 -147.60

contrast estimate SE df t.ratio p.value

Fall - Spring 0.2802 0.0924 187 3.032 0.0078

Fall - Summer 0.2579 0.0917 187 2.812 0.0150

Spring - Summer -0.0223 0.0914 187 -0.244 0.9676

### 21-22 Creatine Kinase

Model selection based on AIC:

K AIC Delta_AIC AICWt Cum.Wt LL

Year 3 3074.12 0.00 0.98 0.98 -1534.06

Sea 4 3083.57 9.45 0.01 0.99 -1537.79

SeaMyco 5 3085.27 11.15 0.00 0.99 -1537.64

null 2 3085.84 11.72 0.00 0.99 -1540.92

Age 3 3086.13 12.01 0.00 1.00 -1540.06

Myco 3 3086.65 12.53 0.00 1.00 -1540.33

Location 5 3087.69 13.57 0.00 1.00 -1538.85

LocMyco 6 3088.10 13.98 0.00 1.00 -1538.05

Sex 4 3088.47 14.35 0.00 1.00 -1540.24

contrast estimate SE df t.ratio p.value

Year2021 - Year2022 562 150 182 3.753 0.0002

### 21-22 AST

Model selection based on AIC:

K AIC Delta_AIC AICWt Cum.Wt LL

Age 3 2143.67 0.00 0.25 0.25 -1068.84

Myco 3 2143.93 0.26 0.22 0.48 -1068.97

SeaMyco 5 2145.12 1.45 0.12 0.60 -1067.56

LocMyco 6 2145.47 1.79 0.10 0.71 -1066.73

null 2 2145.72 2.05 0.09 0.80 -1070.86

Location 5 2145.93 2.26 0.08 0.88 -1067.97

Sea 4 2146.17 2.49 0.07 0.95 -1069.08

Year 3 2147.72 4.05 0.03 0.99 -1070.86

Sex 4 2149.57 5.89 0.01 1.00 -1070.78

contrast estimate SE df t.ratio p.value

Adult - Juvenile -35.5 17.6 189 -2.012 0.0456

### 21-22 Sodium

Model selection based on AIC:

K AIC Delta_AIC AICWt Cum.Wt LL

SeaMyco 5 870.87 0.00 0.68 0.68 -430.43

Sea 4 872.39 1.53 0.32 1.00 -432.20

LocMyco 5 886.82 15.95 0.00 1.00 -438.41

Myco 3 887.11 16.24 0.00 1.00 -440.55

Location 4 893.82 22.95 0.00 1.00 -442.91

null 2 894.37 23.50 0.00 1.00 -445.18

Sex 4 894.84 23.97 0.00 1.00 -443.42

Year 3 895.97 25.11 0.00 1.00 -444.99

Age 3 896.03 25.16 0.00 1.00 -445.01

contrast estimate SE df t.ratio p.value

Fall - Spring 3.36 1.25 134 2.694 0.0216

Fall - Summer -2.61 1.35 134 -1.941 0.1312

Spring - Summer -5.97 1.14 134 -5.223 <.0001

### 21-22 GLDH

Model selection based on AIC:

K AIC Delta_AIC AICWt Cum.Wt LL

SeaMyco 5 1309.10 0.00 0.41 0.41 -649.55

Sea 4 1309.82 0.72 0.29 0.70 -650.91

Sex 4 1311.93 2.83 0.10 0.80 -651.96

null 2 1312.72 3.63 0.07 0.87 -654.36

Myco 3 1312.88 3.79 0.06 0.93 -653.44

Year 3 1314.37 5.27 0.03 0.96 -654.18

Age 3 1314.51 5.42 0.03 0.99 -654.26

LocMyco 6 1317.53 8.44 0.01 0.99 -652.77

Location 5 1317.70 8.60 0.01 1.00 -653.85

contrast estimate SE df t.ratio p.value

Fall - Spring -4.61 1.76 174 -2.615 0.0262

Fall - Summer -1.78 1.77 174 -1.007 0.5735

Spring - Summer 2.83 1.80 174 1.570 0.2613

### 21-22 Glucose

Model selection based on AIC:

K AIC Delta_AIC AICWt Cum.Wt LL

Sea 3 624.33 0.00 0.73 0.73 -309.16

SeaMyco 4 626.32 2.00 0.27 1.00 -309.16

LocMyco 5 657.08 32.75 0.00 1.00 -323.54

Myco 3 659.77 35.45 0.00 1.00 -326.89

Location 4 660.15 35.82 0.00 1.00 -326.08

null 2 661.60 37.28 0.00 1.00 -328.80

Age 3 663.56 39.23 0.00 1.00 -328.78

Sex 4 663.96 39.63 0.00 1.00 -327.98

contrast estimate SE df t.ratio p.value

Spring - Summer -55.8 7.7 62 -7.248 <.0001

### 21-22 Potassium

Model selection based on AIC:

K AIC Delta_AIC AICWt Cum.Wt LL

Sea 3 138.94 0.00 0.69 0.69 -66.47

SeaMyco 4 140.56 1.62 0.31 1.00 -66.28

LocMyco 5 157.93 19.00 0.00 1.00 -73.97

Location 4 158.01 19.08 0.00 1.00 -75.01

null 2 158.64 19.71 0.00 1.00 -77.32

Myco 3 159.60 20.66 0.00 1.00 -76.80

Age 3 160.64 21.71 0.00 1.00 -77.32

Sex 4 162.64 23.70 0.00 1.00 -77.32

contrast estimate SE df t.ratio p.value

Spring - Summer -0.869 0.174 62 -5.003 <.0001

### 22 PCV

Model selection based on AIC:

K AIC Delta_AIC AICWt Cum.Wt LL

null 2 690.49 0.00 0.16 0.16 -343.24

Sea 4 690.82 0.33 0.13 0.29 -341.41

SeaAdeno 5 691.22 0.73 0.11 0.40 -340.61

TrHV1 3 691.26 0.77 0.11 0.51 -342.63

Adeno 3 691.85 1.37 0.08 0.59 -342.93

Myco 3 692.04 1.55 0.07 0.66 -343.02

Location 4 692.05 1.56 0.07 0.73 -342.02

SeaMyco 5 692.07 1.59 0.07 0.80 -341.04

Age 3 692.36 1.87 0.06 0.87 -343.18

SeaTrHV1 5 692.40 1.91 0.06 0.93 -341.20

Sex 4 693.12 2.63 0.04 0.97 -342.56

LocAdeno 5 693.72 3.23 0.03 1.00 -341.86

### 22 TS

Model selection based on AIC:

K AIC Delta_AIC AICWt Cum.Wt LL

Sea 4 277.52 0.00 0.41 0.41 -134.76

SeaMyco 5 278.40 0.88 0.27 0.68 -134.20

SeaAdeno 5 279.39 1.87 0.16 0.84 -134.69

SeaTrHV1 5 279.47 1.95 0.16 1.00 -134.74

TrHV1 3 297.69 20.18 0.00 1.00 -145.85

Age 3 297.70 20.18 0.00 1.00 -145.85

null 2 298.03 20.52 0.00 1.00 -147.02

Location 4 298.68 21.16 0.00 1.00 -145.34

Adeno 3 299.55 22.03 0.00 1.00 -146.78

LocAdeno 5 299.96 22.44 0.00 1.00 -144.98

Myco 3 299.97 22.45 0.00 1.00 -146.99

Sex 4 301.17 23.65 0.00 1.00 -146.58

contrast estimate SE df t.ratio p.value

Fall - Spring 0.215 0.225 99 0.956 0.6066

Fall - Summer 1.084 0.222 99 4.893 <.0001

Spring - Summer 0.870 0.223 99 3.893 0.0005

### 22 WBC

Model selection based on AIC:

K AIC Delta_AIC AICWt Cum.Wt LL

Sea 4 2122.88 0.00 0.39 0.39 -1057.44

SeaTrHV1 5 2123.80 0.92 0.24 0.63 -1056.90

SeaAdeno 5 2124.05 1.17 0.22 0.85 -1057.02

SeaMyco 5 2124.72 1.83 0.15 1.00 -1057.36

Age 3 2145.83 22.94 0.00 1.00 -1069.91

Sex 4 2149.05 26.17 0.00 1.00 -1070.53

Myco 3 2149.30 26.41 0.00 1.00 -1071.65

null 2 2152.27 29.38 0.00 1.00 -1074.13

TrHV1 3 2152.88 30.00 0.00 1.00 -1073.44

Adeno 3 2154.04 31.16 0.00 1.00 -1074.02

Location 4 2155.59 32.71 0.00 1.00 -1073.80

LocAdeno 5 2157.25 34.37 0.00 1.00 -1073.63

contrast estimate SE df t.ratio p.value

Fall - Spring 11786 1908 99 6.176 <.0001

Fall - Summer 5099 1880 99 2.712 0.0213

Spring - Summer -6687 1895 99 -3.529 0.0018

### 22 ESR

Model selection based on AIC:

K AIC Delta_AIC AICWt Cum.Wt LL

SeaTrHV1 5 506.25 0.00 0.64 0.64 -248.12

Sea 4 508.59 2.35 0.20 0.84 -250.30

SeaAdeno 5 510.37 4.12 0.08 0.92 -250.18

SeaMyco 5 510.55 4.31 0.07 1.00 -250.28

TrHV1 3 517.39 11.14 0.00 1.00 -255.69

null 2 522.75 16.50 0.00 1.00 -259.38

Adeno 3 523.15 16.91 0.00 1.00 -258.58

Age 3 524.48 18.24 0.00 1.00 -259.24

Myco 3 524.61 18.36 0.00 1.00 -259.30

Sex 4 525.83 19.58 0.00 1.00 -258.91

LocAdeno 5 526.61 20.36 0.00 1.00 -258.30

Location 4 526.65 20.40 0.00 1.00 -259.32

contrast estimate SE df t.ratio p.value

Neg - Pos 1.7 0.825 98 2.065 0.0415

contrast estimate SE df t.ratio p.value

Fall - Spring -1.930 0.688 98 -2.807 0.0165

Fall - Summer -2.645 0.698 98 -3.790 0.0008

Spring - Summer -0.715 0.711 98 -1.005 0.5752

### 22 Heterophils

Model selection based on AIC:

K AIC Delta_AIC AICWt Cum.Wt LL

Sea 4 1712.40 0.00 0.41 0.41 -852.20

SeaAdeno 5 1713.60 1.20 0.23 0.64 -851.80

SeaTrHV1 5 1713.75 1.36 0.21 0.84 -851.88

SeaMyco 5 1714.35 1.95 0.16 1.00 -852.17

Myco 3 1729.45 17.05 0.00 1.00 -861.72

null 2 1731.16 18.77 0.00 1.00 -863.58

TrHV1 3 1731.57 19.18 0.00 1.00 -862.79

Location 4 1731.94 19.54 0.00 1.00 -861.97

Age 3 1732.38 19.99 0.00 1.00 -863.19

Adeno 3 1732.53 20.13 0.00 1.00 -863.26

LocAdeno 5 1733.69 21.29 0.00 1.00 -861.84

Sex 4 1733.84 21.45 0.00 1.00 -862.92

contrast estimate SE df t.ratio p.value

Fall - Spring 1215 255 99 4.760 <.0001

Fall - Summer 283 251 99 1.125 0.5012

Spring - Summer -932 253 99 -3.678 0.0011

### 22 Lymphocytes

Model selection based on AIC:

K AIC Delta_AIC AICWt Cum.Wt LL

Sea 4 2020.52 0.00 0.35 0.35 -1006.26

SeaAdeno 5 2020.70 0.18 0.32 0.66 -1005.35

SeaTrHV1 5 2021.83 1.31 0.18 0.84 -1005.92

SeaMyco 5 2022.27 1.74 0.14 0.99 -1006.13

Age 3 2027.52 7.00 0.01 1.00 -1010.76

Myco 3 2031.99 11.46 0.00 1.00 -1012.99

Sex 4 2032.12 11.60 0.00 1.00 -1012.06

null 2 2033.66 13.14 0.00 1.00 -1014.83

TrHV1 3 2034.42 13.90 0.00 1.00 -1014.21

Adeno 3 2034.46 13.93 0.00 1.00 -1014.23

Location 4 2037.66 17.13 0.00 1.00 -1014.83

LocAdeno 5 2038.37 17.85 0.00 1.00 -1014.19

contrast estimate SE df t.ratio p.value

Fall - Spring 4833 1155 99 4.183 0.0002

Fall - Summer 1606 1139 99 1.411 0.3394

Spring - Summer -3227 1147 99 -2.813 0.0162

### 22 Eosinophils

Model selection based on AIC:

K AIC Delta_AIC AICWt Cum.Wt LL

Sea 4 1784.09 0.00 0.37 0.37 -888.04

SeaTrHV1 5 1784.18 0.09 0.35 0.73 -887.09

SeaMyco 5 1786.06 1.97 0.14 0.86 -888.03

SeaAdeno 5 1786.08 2.00 0.14 1.00 -888.04

Sex 4 1818.75 34.67 0.00 1.00 -905.38

Age 3 1820.64 36.56 0.00 1.00 -907.32

Myco 3 1821.11 37.03 0.00 1.00 -907.56

null 2 1824.08 40.00 0.00 1.00 -910.04

TrHV1 3 1824.14 40.05 0.00 1.00 -909.07

Location 4 1825.35 41.26 0.00 1.00 -908.67

Adeno 3 1825.99 41.91 0.00 1.00 -910.00

LocAdeno 5 1827.22 43.13 0.00 1.00 -908.61

contrast estimate SE df t.ratio p.value

Fall - Spring 2646 363 99 7.299 <.0001

Fall - Summer 1199 357 99 3.357 0.0032

Spring - Summer -1447 360 99 -4.019 0.0003

### 22 Basophils

Model selection based on AIC:

K AIC Delta_AIC AICWt Cum.Wt LL

Sea 4 1848.79 0.00 0.43 0.43 -920.40

SeaAdeno 5 1850.01 1.22 0.23 0.66 -920.00

SeaTrHV1 5 1850.46 1.67 0.19 0.84 -920.23

SeaMyco 5 1850.78 1.99 0.16 1.00 -920.39

Age 3 1873.12 24.33 0.00 1.00 -933.56

Sex 4 1875.28 26.49 0.00 1.00 -933.64

Myco 3 1877.49 28.70 0.00 1.00 -935.74

null 2 1878.31 29.52 0.00 1.00 -937.16

TrHV1 3 1880.14 31.35 0.00 1.00 -937.07

Adeno 3 1880.28 31.48 0.00 1.00 -937.14

Location 4 1880.34 31.55 0.00 1.00 -936.17

LocAdeno 5 1882.19 33.40 0.00 1.00 -936.10

contrast estimate SE df t.ratio p.value

Fall - Spring 3044 498 99 6.115 <.0001

Fall - Summer 1951 491 99 3.976 0.0004

Spring - Summer -1094 494 99 -2.213 0.0739

### 22 HL ratio

| Model selection based on AIC:  K AIC Delta_AIC AICWt Cum.Wt LL  Sea 4 -10.93 0.00 0.28 0.28 9.46  SeaAdeno 5 -10.83 0.09 0.26 0.54 10.42  SeaMyco 5 -9.14 1.78 0.11 0.65 9.57  SeaTrHV1 5 -8.94 1.99 0.10 0.76 9.47  Adeno 3 -7.71 3.22 0.06 0.81 6.85  Age 3 -7.26 3.67 0.04 0.86 6.63  null 2 -6.97 3.96 0.04 0.89 5.48  LocAdeno 5 -6.75 4.18 0.03 0.93 8.38  Location 4 -6.44 4.48 0.03 0.96 7.22  Myco 3 -5.07 5.86 0.01 0.97 5.53  TrHV1 3 -4.97 5.96 0.01 0.99 5.48  Sex 4 -4.94 5.99 0.01 1.00 6.47  contrast estimate SE df t.ratio p.value  Fall - Spring 0.147 0.0547 99 2.696 0.0223  Fall - Summer 0.114 0.0539 99 2.106 0.0937  Spring - Summer -0.034 0.0543 99 -0.625 0.8066 |
| --- |

### 22 Total calcium

Model selection based on AIC:

K AIC Delta_AIC AICWt Cum.Wt LL

SexAge 5 485.87 0.00 0.53 0.53 -237.93

Sex 4 487.03 1.17 0.30 0.83 -239.52

Sea 4 490.40 4.53 0.06 0.88 -241.20

SeaAdeno 5 490.82 4.95 0.04 0.93 -240.41

SeaMyco 5 491.56 5.70 0.03 0.96 -240.78

SeaTrHV1 5 492.06 6.20 0.02 0.98 -241.03

Myco 3 494.76 8.89 0.01 0.99 -244.38

Adeno 3 496.51 10.64 0.00 0.99 -245.25

Location 4 496.60 10.73 0.00 0.99 -244.30

null 2 496.78 10.91 0.00 1.00 -246.39

LocAdeno 5 497.49 11.63 0.00 1.00 -243.75

Age 3 497.91 12.05 0.00 1.00 -245.96

TrHV1 3 498.63 12.76 0.00 1.00 -246.31

contrast estimate SE df t.ratio p.value

Female - Male 2.155 0.57 96 3.779 0.0008

Female - Unknown 0.933 1.30 96 0.717 0.7541

Male - Unknown -1.221 1.30 96 -0.942 0.6152

### 22 Phosphorus

Model selection based on AIC:

K AIC Delta_AIC AICWt Cum.Wt LL

Sea 4 248.14 0.00 0.35 0.35 -120.07

SeaMyco 5 249.08 0.94 0.22 0.58 -119.54

SeaAdeno 5 249.13 0.99 0.22 0.79 -119.57

SeaTrHV1 5 249.19 1.05 0.21 1.00 -119.59

Sex 4 314.53 66.40 0.00 1.00 -153.27

TrHV1 3 317.22 69.09 0.00 1.00 -155.61

Myco 3 319.22 71.08 0.00 1.00 -156.61

LocAdeno 5 319.74 71.60 0.00 1.00 -154.87

Age 3 322.25 74.11 0.00 1.00 -158.12

Adeno 3 322.82 74.68 0.00 1.00 -158.41

Location 4 322.84 74.70 0.00 1.00 -157.42

null 2 324.87 76.73 0.00 1.00 -160.43

contrast estimate SE df t.ratio p.value

Fall - Spring 0.565 0.203 97 2.792 0.0172

Fall - Summer -1.529 0.200 97 -7.661 <.0001

Spring - Summer -2.095 0.198 97 -10.577 <.0001

### 22 CaP ratio

Model selection based on AIC:

K AIC Delta_AIC AICWt Cum.Wt LL

SeaTrHV1 5 284.62 0.00 0.49 0.49 -137.31

Sea 4 285.73 1.10 0.28 0.77 -138.86

SeaAdeno 5 287.52 2.90 0.11 0.89 -138.76

SeaMyco 5 287.53 2.90 0.11 1.00 -138.76

TrHV1 3 311.45 26.82 0.00 1.00 -152.72

Age 3 315.02 30.40 0.00 1.00 -154.51

Myco 3 319.59 34.97 0.00 1.00 -156.80

Adeno 3 320.28 35.66 0.00 1.00 -157.14

null 2 320.41 35.79 0.00 1.00 -158.20

LocAdeno 5 320.64 36.02 0.00 1.00 -155.32

Sex 4 321.51 36.89 0.00 1.00 -156.76

Location 4 322.09 37.47 0.00 1.00 -157.05

contrast estimate SE df t.ratio p.value

Fall - Spring -0.157 0.250 96 -0.630 0.8039

Fall - Summer 1.338 0.244 96 5.476 <.0001

Spring - Summer 1.495 0.244 96 6.120 <.0001

### 22 Bile acids

Model selection based on AIC:

K AIC Delta_AIC AICWt Cum.Wt LL

Age 3 759.15 0.00 0.82 0.82 -376.58

Sex 4 762.16 3.00 0.18 1.00 -377.08

LocAdeno 5 774.26 15.11 0.00 1.00 -382.13

Adeno 3 774.48 15.33 0.00 1.00 -384.24

SeaAdeno 5 776.82 17.67 0.00 1.00 -383.41

null 2 778.03 18.88 0.00 1.00 -387.01

Sea 4 779.17 20.02 0.00 1.00 -385.59

Myco 3 779.80 20.65 0.00 1.00 -386.90

TrHV1 3 779.89 20.74 0.00 1.00 -386.94

Location 4 780.27 21.11 0.00 1.00 -386.13

SeaTrHV1 5 781.14 21.98 0.00 1.00 -385.57

SeaMyco 5 781.15 21.99 0.00 1.00 -385.57

contrast estimate SE df t.ratio p.value

Adult - Juvenile -24 5.03 97 -4.772 <.0001

### 22 Uric acid

Model selection based on AIC:

K AIC Delta_AIC AICWt Cum.Wt LL

null 2 87.90 0.00 0.19 0.19 -41.95

Location 4 87.95 0.05 0.18 0.37 -39.97

Myco 3 89.03 1.13 0.11 0.47 -41.51

Age 3 89.08 1.18 0.10 0.58 -41.54

Adeno 3 89.23 1.34 0.10 0.67 -41.62

TrHV1 3 89.34 1.45 0.09 0.76 -41.67

LocAdeno 5 89.86 1.96 0.07 0.83 -39.93

Sea 4 90.29 2.39 0.06 0.89 -41.15

SeaTrHV1 5 91.62 3.72 0.03 0.92 -40.81

Sex 4 91.67 3.77 0.03 0.95 -41.83

SeaAdeno 5 91.81 3.92 0.03 0.97 -40.91

SeaMyco 5 91.88 3.99 0.03 1.00 -40.94

contrast estimate SE df t.ratio p.value

Fall - Spring 0.1125 0.0931 96 1.209 0.4510

Fall - Summer 0.0821 0.0911 96 0.901 0.6410

Spring - Summer -0.0304 0.0911 96 -0.334 0.9403

### 22 Creatine Kinase

Model selection based on AIC:

K AIC Delta_AIC AICWt Cum.Wt LL

SeaAdeno 5 1454.04 0.00 0.54 0.54 -722.02

Sea 4 1456.49 2.45 0.16 0.69 -724.24

SeaMyco 5 1457.64 3.60 0.09 0.78 -723.82

SeaTrHV1 5 1457.77 3.74 0.08 0.87 -723.89

TrHV1 3 1459.63 5.59 0.03 0.90 -726.81

Myco 3 1459.83 5.79 0.03 0.93 -726.92

null 2 1460.18 6.14 0.02 0.95 -728.09

Adeno 3 1460.43 6.39 0.02 0.97 -727.21

Age 3 1462.17 8.14 0.01 0.98 -728.09

Sex 4 1462.72 8.68 0.01 0.99 -727.36

Location 4 1463.12 9.08 0.01 1.00 -727.56

LocAdeno 5 1463.95 9.92 0.00 1.00 -726.98

contrast estimate SE df t.ratio p.value

Neg - Pos 303 145 90 2.089 0.0396

contrast estimate SE df t.ratio p.value

Fall - Spring 60.7 134 90 0.452 0.8935

Fall - Summer -361.6 141 90 -2.573 0.0312

Spring - Summer -422.4 139 90 -3.048 0.0084

### 22 AST

Model selection based on AIC:

K AIC Delta_AIC AICWt Cum.Wt LL

Age 3 1129.39 0.00 0.19 0.19 -561.69

null 2 1129.84 0.45 0.15 0.35 -562.92

Myco 3 1130.33 0.95 0.12 0.47 -562.17

Location 4 1130.51 1.12 0.11 0.58 -561.26

TrHV1 3 1130.63 1.24 0.10 0.68 -562.31

Sex 4 1130.89 1.50 0.09 0.77 -561.44

Adeno 3 1131.74 2.35 0.06 0.83 -562.87

Sea 4 1132.11 2.73 0.05 0.88 -562.06

LocAdeno 5 1132.41 3.02 0.04 0.92 -561.21

SeaMyco 5 1133.11 3.72 0.03 0.95 -561.55

SeaTrHV1 5 1133.60 4.21 0.02 0.98 -561.80

SeaAdeno 5 1133.77 4.38 0.02 1.00 -561.88

contrast estimate SE df t.ratio p.value

Adult - Juvenile -48.1 30.8 98 -1.560 0.1220

### 22 Sodium

Model selection based on AIC:

K AIC Delta_AIC AICWt Cum.Wt LL

SeaMyco 5 555.78 0.00 0.77 0.77 -272.89

Sea 4 560.01 4.23 0.09 0.87 -276.00

SeaTrHV1 5 560.16 4.39 0.09 0.95 -275.08

SeaAdeno 5 561.46 5.69 0.05 1.00 -275.73

Myco 3 582.09 26.31 0.00 1.00 -288.05

Location 4 591.67 35.89 0.00 1.00 -291.83

Sex 4 592.93 37.16 0.00 1.00 -292.47

LocAdeno 5 593.26 37.49 0.00 1.00 -291.63

Age 3 595.60 39.83 0.00 1.00 -294.80

null 2 596.61 40.83 0.00 1.00 -296.30

Adeno 3 596.77 40.99 0.00 1.00 -295.38

TrHV1 3 598.25 42.47 0.00 1.00 -296.12

contrast estimate SE df t.ratio p.value

Neg - Pos 3.88 1.56 97 2.484 0.0147

contrast estimate SE df t.ratio p.value

Fall - Spring 3.03 0.962 97 3.153 0.0060

Fall - Summer -2.46 0.900 97 -2.733 0.0202

Spring - Summer -5.49 0.943 97 -5.826 <.0001

### 22 GLDH

Model selection based on AIC:

K AIC Delta_AIC AICWt Cum.Wt LL

Sea 4 638.63 0.00 0.26 0.26 -315.32

SeaMyco 5 639.16 0.52 0.20 0.46 -314.58

SeaTrHV1 5 640.12 1.49 0.12 0.59 -315.06

SeaAdeno 5 640.63 2.00 0.10 0.68 -315.31

Myco 3 641.19 2.56 0.07 0.76 -317.59

TrHV1 3 641.32 2.69 0.07 0.83 -317.66

null 2 641.47 2.84 0.06 0.89 -318.74

Location 4 642.57 3.93 0.04 0.93 -317.28

Adeno 3 643.23 4.60 0.03 0.95 -318.62

Age 3 643.43 4.79 0.02 0.98 -318.71

LocAdeno 5 644.36 5.73 0.01 0.99 -317.18

Sex 4 645.38 6.75 0.01 1.00 -318.69

contrast estimate SE df t.ratio p.value

Fall - Spring -0.397 1.53 95 -0.259 0.9638

Fall - Summer -3.594 1.51 95 -2.378 0.0503

Spring - Summer -3.197 1.51 95 -2.116 0.0921

### 22 Glucose

Model selection based on AIC:

K AIC Delta_AIC AICWt Cum.Wt LL

Sea 3 669.99 0.00 0.45 0.45 -332.00

SeaTrHV1 4 671.53 1.54 0.21 0.66 -331.77

SeaAdeno 4 671.85 1.86 0.18 0.83 -331.92

SeaMyco 4 671.99 1.99 0.17 1.00 -331.99

TrHV1 3 704.32 34.32 0.00 1.00 -349.16

Myco 3 707.79 37.80 0.00 1.00 -350.90

Location 4 708.44 38.44 0.00 1.00 -350.22

null 2 709.78 39.78 0.00 1.00 -352.89

LocAdeno 5 709.84 39.85 0.00 1.00 -349.92

Adeno 3 710.28 40.29 0.00 1.00 -352.14

Sex 4 711.22 41.22 0.00 1.00 -351.61

Age 3 711.73 41.74 0.00 1.00 -352.87

contrast estimate SE df t.ratio p.value

Spring - Summer -54.3 7.27 67 -7.467 <.0001

### 22 Potassium

Model selection based on AIC:

K AIC Delta_AIC AICWt Cum.Wt LL

Sea 3 186.01 0.00 0.45 0.45 -90.00

SeaTrHV1 4 187.60 1.59 0.20 0.65 -89.80

SeaAdeno 4 187.86 1.86 0.18 0.83 -89.93

SeaMyco 4 187.97 1.97 0.17 1.00 -89.99

TrHV1 3 197.48 11.47 0.00 1.00 -95.74

Location 4 199.15 13.15 0.00 1.00 -95.58

null 2 199.55 13.54 0.00 1.00 -97.78

Myco 3 200.28 14.28 0.00 1.00 -97.14

LocAdeno 5 201.12 15.11 0.00 1.00 -95.56

Adeno 3 201.46 15.46 0.00 1.00 -97.73

Age 3 201.52 15.51 0.00 1.00 -97.76

Sex 4 202.99 16.99 0.00 1.00 -97.50

contrast estimate SE df t.ratio p.value

Spring - Summer -0.897 0.218 67 -4.115 0.0001

### EPH TP

Model selection based on AIC:

K AIC Delta_AIC AICWt Cum.Wt LL

Sea 4 275.52 0.00 0.41 0.41 -133.76

SeaMyco 5 276.39 0.87 0.27 0.68 -133.20

SeaAdeno 5 277.37 1.85 0.16 0.84 -133.68

SeaTrHV1 5 277.46 1.95 0.16 1.00 -133.73

Age 3 294.73 19.22 0.00 1.00 -144.37

TrHV1 3 295.04 19.52 0.00 1.00 -144.52

null 2 295.61 20.10 0.00 1.00 -145.81

Location 4 295.84 20.32 0.00 1.00 -143.92

LocAdeno 5 297.17 21.66 0.00 1.00 -143.59

Adeno 3 297.20 21.68 0.00 1.00 -145.60

Myco 3 297.56 22.05 0.00 1.00 -145.78

Sex 4 298.66 23.15 0.00 1.00 -145.33

contrast estimate SE df t.ratio p.value

Fall - Spring 0.203 0.227 98 0.894 0.6455

Fall - Summer 1.082 0.226 98 4.790 <.0001

Spring - Summer 0.879 0.222 98 3.951 0.0004

### EPH AG ratio

Model selection based on AIC:

K AIC Delta_AIC AICWt Cum.Wt LL

Age 3 -173.88 0.00 0.72 0.72 89.94

Sex 4 -169.99 3.89 0.10 0.82 89.00

null 2 -168.49 5.39 0.05 0.87 86.25

Sea 4 -166.83 7.06 0.02 0.89 87.41

TrHV1 3 -166.58 7.30 0.02 0.91 86.29

Myco 3 -166.57 7.31 0.02 0.93 86.29

Adeno 3 -166.53 7.35 0.02 0.95 86.27

Location 4 -166.51 7.38 0.02 0.97 87.25

SeaAdeno 5 -164.99 8.89 0.01 0.98 87.50

LocAdeno 5 -164.91 8.97 0.01 0.98 87.46

SeaMyco 5 -164.91 8.98 0.01 0.99 87.45

SeaTrHV1 5 -164.85 9.04 0.01 1.00 87.42

contrast estimate SE df t.ratio p.value

Adult - Juvenile -0.126 0.046 99 -2.742 0.0073

### EPH PreAlb

Model selection based on AIC:

K AIC Delta_AIC AICWt Cum.Wt LL

Location 4 -465.15 0.00 0.72 0.72 236.57

LocAdeno 5 -463.30 1.85 0.28 1.00 236.65

SeaMyco 5 -445.31 19.84 0.00 1.00 227.65

Sea 4 -442.47 22.67 0.00 1.00 225.24

SeaTrHV1 5 -440.51 24.63 0.00 1.00 225.26

SeaAdeno 5 -440.50 24.64 0.00 1.00 225.25

Sex 4 -436.00 29.14 0.00 1.00 222.00

null 2 -435.29 29.86 0.00 1.00 219.64

TrHV1 3 -434.72 30.43 0.00 1.00 220.36

Myco 3 -434.12 31.03 0.00 1.00 220.06

Adeno 3 -433.45 31.70 0.00 1.00 219.72

Age 3 -433.31 31.83 0.00 1.00 219.66

contrast estimate SE df t.ratio p.value

Burnham - Park Ridge -0.058077 0.00950 98 -6.110 <.0001

Burnham - Powderhorn -0.000696 0.00490 98 -0.142 0.9889

Park Ridge - Powderhorn 0.057381 0.00964 98 5.954 <.0001

### EPH Albumin

Model selection based on AIC:

K AIC Delta_AIC AICWt Cum.Wt LL

Sea 4 4.14 0.00 0.43 0.43 1.93

SeaTrHV1 5 5.50 1.37 0.22 0.65 2.25

SeaMyco 5 5.80 1.66 0.19 0.84 2.10

SeaAdeno 5 6.12 1.98 0.16 1.00 1.94

TrHV1 3 20.68 16.54 0.00 1.00 -7.34

LocAdeno 5 22.45 18.31 0.00 1.00 -6.22

null 2 22.49 18.36 0.00 1.00 -9.25

Location 4 22.52 18.38 0.00 1.00 -7.26

Adeno 3 23.81 19.67 0.00 1.00 -8.90

Sex 4 24.09 19.96 0.00 1.00 -8.05

Age 3 24.33 20.20 0.00 1.00 -9.17

Myco 3 24.48 20.35 0.00 1.00 -9.24

contrast estimate SE df t.ratio p.value

Fall - Spring 0.0955 0.0594 98 1.610 0.2465

Fall - Summer 0.2839 0.0589 98 4.817 <.0001

Spring - Summer 0.1884 0.0580 98 3.246 0.0045

### EPH Alpha 1

Model selection based on AIC:

K AIC Delta_AIC AICWt Cum.Wt LL

Sea 4 -260.30 0.00 0.44 0.44 134.15

SeaMyco 5 -258.72 1.58 0.20 0.64 134.36

SeaTrHV1 5 -258.54 1.77 0.18 0.82 134.27

SeaAdeno 5 -258.45 1.85 0.17 1.00 134.23

TrHV1 3 -245.98 14.32 0.00 1.00 125.99

null 2 -245.59 14.71 0.00 1.00 124.79

Location 4 -245.43 14.87 0.00 1.00 126.71

Age 3 -244.20 16.10 0.00 1.00 125.10

LocAdeno 5 -244.18 16.12 0.00 1.00 127.09

Adeno 3 -243.86 16.44 0.00 1.00 124.93

Myco 3 -243.69 16.61 0.00 1.00 124.85

Sex 4 -242.81 17.49 0.00 1.00 125.41

contrast estimate SE df t.ratio p.value

Fall - Spring 0.0281 0.0160 98 1.753 0.1909

Fall - Summer 0.0704 0.0159 98 4.422 0.0001

Spring - Summer 0.0423 0.0157 98 2.698 0.0222

### EPH Alpha 2

Model selection based on AIC:

K AIC Delta_AIC AICWt Cum.Wt LL

Sea 4 -54.33 0.00 0.43 0.43 31.17

SeaMyco 5 -52.86 1.48 0.21 0.64 31.43

SeaAdeno 5 -52.72 1.62 0.19 0.83 31.36

SeaTrHV1 5 -52.42 1.91 0.17 1.00 31.21

Location 4 -14.90 39.43 0.00 1.00 11.45

LocAdeno 5 -13.63 40.70 0.00 1.00 11.82

TrHV1 3 -6.78 47.55 0.00 1.00 6.39

Age 3 -6.28 48.06 0.00 1.00 6.14

Myco 3 -5.73 48.60 0.00 1.00 5.86

null 2 -5.57 48.76 0.00 1.00 4.78

Adeno 3 -3.94 50.39 0.00 1.00 4.97

Sex 4 -2.58 51.75 0.00 1.00 5.29

contrast estimate SE df t.ratio p.value

Fall - Spring -0.138 0.0444 98 -3.116 0.0067

Fall - Summer 0.215 0.0441 98 4.868 <.0001

Spring - Summer 0.353 0.0434 98 8.131 <.0001

### EPH Beta

Model selection based on AIC:

K AIC Delta_AIC AICWt Cum.Wt LL

Sex 4 78.57 0.00 0.57 0.57 -35.29

Sea 4 82.13 3.56 0.10 0.66 -37.06

SeaMyco 5 82.73 4.16 0.07 0.73 -36.36

Myco 3 83.27 4.70 0.05 0.79 -38.64

null 2 83.49 4.91 0.05 0.83 -39.74

Age 3 83.59 5.02 0.05 0.88 -38.80

SeaAdeno 5 84.13 5.56 0.04 0.92 -37.06

SeaTrHV1 5 84.13 5.56 0.04 0.95 -37.06

Adeno 3 85.35 6.78 0.02 0.97 -39.68

TrHV1 3 85.41 6.84 0.02 0.99 -39.71

Location 4 86.87 8.30 0.01 1.00 -39.43

LocAdeno 5 88.85 10.28 0.00 1.00 -39.42

contrast estimate SE df t.ratio p.value

Female - Male 0.2134 0.0712 98 2.996 0.0096

Female - Unknown 0.0716 0.1642 98 0.436 0.9008

Male - Unknown -0.1419 0.1633 98 -0.869 0.6610

### EPH Gamma

Model selection based on AIC:

K AIC Delta_AIC AICWt Cum.Wt LL

Sea 4 27.26 0.00 0.43 0.43 -9.63

SeaMyco 5 28.73 1.46 0.21 0.64 -9.36

SeaAdeno 5 28.84 1.57 0.20 0.84 -9.42

SeaTrHV1 5 29.24 1.97 0.16 1.00 -9.62

Age 3 45.33 18.07 0.00 1.00 -19.67

TrHV1 3 48.35 21.08 0.00 1.00 -21.17

Location 4 48.79 21.53 0.00 1.00 -20.39

null 2 48.91 21.65 0.00 1.00 -22.46

Sex 4 50.07 22.81 0.00 1.00 -21.04

LocAdeno 5 50.42 23.16 0.00 1.00 -20.21

Adeno 3 50.72 23.46 0.00 1.00 -22.36

Myco 3 50.90 23.64 0.00 1.00 -22.45

contrast estimate SE df t.ratio p.value

Fall - Spring 0.0517 0.0666 98 0.777 0.7182

Fall - Summer 0.3242 0.0661 98 4.905 <.0001

Spring - Summer 0.2725 0.0651 98 4.188 0.0002

### Mycoplasma detection

Model selection based on AIC:

K AIC Delta_AIC AICWt Cum.Wt LL

SeaLoc 4 103.34 0.00 0.98 0.98 -47.67

Sea 3 111.53 8.19 0.02 1.00 -52.76

Loc 2 116.42 13.07 0.00 1.00 -56.21

Sex 3 123.45 20.11 0.00 1.00 -58.72

null 1 123.76 20.42 0.00 1.00 -60.88

Yea 2 124.60 21.26 0.00 1.00 -60.30

SexAge 4 125.15 21.81 0.00 1.00 -58.58

Age 2 125.47 22.13 0.00 1.00 -60.73

contrast odds.ratio SE df null z.ratio p.value

Fall / Spring 1 1.00e+00 Inf 1 -0.119 0.9922

Fall / Summer 49718175 6.19e+10 Inf 1 0.014 0.9999

Spring / Summer 53041747 6.61e+10 Inf 1 0.014 0.9999

contrast odds.ratio SE df null z.ratio p.value

Other / Powderhorn 0.127 0.0995 Inf 1 -2.633 0.0085

### TrHV1 detection

Model selection based on AIC:

K AIC Delta_AIC AICWt Cum.Wt LL

Sea 3 80.93 0.00 0.95 0.95 -37.47

SeaLocSexAge 8 87.40 6.47 0.04 0.98 -35.70

Sex 3 91.29 10.36 0.01 0.99 -42.65

Age 2 91.58 10.65 0.00 0.99 -43.79

null 1 91.63 10.70 0.00 1.00 -44.82

Loc 3 94.81 13.88 0.00 1.00 -44.40

contrast odds.ratio SE df null z.ratio p.value

Fall / Spring 1.00e+00 0.00e+00 Inf 1 -0.568 0.5700

Fall / Summer 7.86e+07 1.43e+11 Inf 1 0.010 0.9920

Spring / Summer 1.09e+08 1.98e+11 Inf 1 0.010 0.9919
